# ISGylation enhances dsRNA-induced interferon response and NFκB signaling in fallopian tube epithelial cells

**DOI:** 10.1016/j.jbc.2024.107686

**Published:** 2024-08-17

**Authors:** Vidushi Madaan, Alexandra Kollara, David Spaner, Theodore J. Brown

**Affiliations:** 1 Lunenfeld-Tanenbaum Research Institute, Sinai Health, Toronto, Ontario, Canada; 2 Institute of Medical Science, University of Toronto, Toronto, Ontario, Canada; 3 Sunnybrook Research Institute, Sunnybrook Health Sciences Centre, Toronto, Ontario, Canada; 4 Department of Obstetrics and Gynaecology, University of Toronto, Toronto, Ontario, Canada

**Keywords:** ovarian cancer, fallopian tube cells, ISGylation, dsRNA, inflammation, CRISPR/cas, innate immunity, RIGI-like receptor, interferon, NF-kappa B

## Abstract

Heritable mutations in *BRCA1* associate with increased risk of high-grade serous tubo-ovarian cancer. Nongenetic risk factors associated with this cancer, which arises from fallopian tube epithelial (FTE) cells, suggests a role for repetitive ovulation wherein FTE cells are exposed to inflammatory signaling molecules within follicular fluid. We previously reported increased NFκB and EGFR signaling in BRCA1-deficient primary FTE cells, with follicular fluid exposure further increasing abundance of interferon-stimulated gene (ISG) transcripts, including the ubiquitin-like protein ISG15 and other ISGylation pathway members. Both NFκB and type I interferon signaling are upregulated by stimulation of cGAS-STING or MDA5 and RIGI pattern recognition receptors. Since some pattern recognition receptors and their signal transduction pathway members are ISGylated, we tested the impact of ISG15 and ISGylation on interferon regulatory factor 3 (IRF3) and NFκB signaling through cGAS-STING or RIGI and MDA5 activation. Expression of *ISG15* or *UBA7*, the E1-like ISG15-activating enzyme, in immortalized FTE cells was disrupted by CRISPR gene editing. Activation of IRF3 by RIGI or MDA5 but not cGAS-STING was attenuated by loss of either ISG15 or UBA7 and this was reflected by a similar effect on NFκB activation and downstream targets. Loss of ISGylation decreased levels of both MDA5 and RIGI, with knockdown of RIGI but not MDA5, decreasing IRF3 and NFκB activation in parental cells. These finding indicate that ISGylation enhances the ability of dsRNA to activate cytokine release and proinflammatory signaling. Further work to explore ISGylation as a target for prevention of high-grade serous tubo-ovarian cancer in *BRCA1* mutation carriers is warranted.

High-grade serous tubo-ovarian cancer (HGSTOC) is the most commonly diagnosed form of ovarian cancer in Western countries (1, 2). As this cancer is largely asymptomatic at early stages, it typically presents at an advanced stage, with a median survival of less than 4 years (3). Precursor lesions for HGSTOC have been identified within the fallopian tube epithelium (FTE) (4) and existing serum biomarkers have not proven to be sufficiently sensitive or specific for detection of early stage disease. The lifetime incidence of ovarian cancer in the general female population is estimated at 1.3% (2); however, individuals with germ-line mutations in *BRCA1* have an incidence of HGSTOC of up to 44% (5, 6). Moreover, these individuals have an earlier age of onset as compared to spontaneous cases or to cases with mutations in other identified susceptibility genes (7, 8). Because of this high risk, prophylactic bilateral salpingo-oophorectomy is recommended to these individuals between 35 and 40 years of age or 5 to 10 years prior to the earliest age of diagnosis in affected family members (9). Nevertheless, many delay this procedure to complete child bearing. Identification of targets for prevention of HGSTOC based upon the understanding of factors promoting HGSTOC initiation and early progression are needed for identified predisposed individuals.

A decreased risk for HGSTOC is associated with oral contraceptive use, lactation, and pregnancy. Since a shared action of these three factors is the inhibition of ovulation, a prevailing hypothesis is that repetitive exposure of FTE cells to proinflammatory factors within follicular fluid increases the risk of malignant transformation (10, 11, 12). We previously demonstrated increased NFκB and EGFR signaling in FTE cells from *BRCA1* mutation carriers relative to FTE cells from nonmutation carriers (13, 14, 15). Exposure to periovulatory follicular fluid further increased proinflammatory signaling and transcript levels of interferon-stimulated genes (ISGs), including ISG15 and ISGylation pathway members (13). ISG15 is an ubiquitin-like protein that can act as a free intracellular or secreted protein or can covalently conjugate to client proteins through the process of ISGylation to modulate their activity (reviewed in (16)). Similar to ubiquitination, ISGylation is a reversible process involving three enzymatic steps, whereby a single ISG15 molecule is attached to a target protein: ISG15 activation (E1), ISG15 conjugation (E2), and ISG15 ligation (E3). *UBA7/UBE1L* encodes the only ISG15-activating enzyme identified to date and functions in the first of the three steps of the ISGylation pathway. Removal of ISG15 from target proteins involves the isopeptidase activity of USP18. In addition, USP18, which is increased by interferon signaling and is stabilized through a noncovalent association with ISG15, also acts to inhibit STAT1 activation by type I interferons to limit undue interferon signaling (17, 18).

Interferon and downstream ISGs can be upregulated by activation of intracellular DNA or RNA pattern recognition receptors such as cGAS-STING or MDA5 and RIGI. These cytoplasmic receptors are part of the innate immune system and recognize aberrant nucleic acids in cells that can accumulate due to BRCA1 insufficiency (19, 20, 21). Stimulation of either cGAS-STING or MDA5-MAVS and RIGI-MAVS activates both interferon regulatory factor 3 (IRF3), a key driver of type I interferon expression, and NFκB signaling (22, 23).

Several members of the pattern recognition receptor signaling network have been shown to be ISGylated, including MDA5, RIGI, STING, and IRF3 (24, 25, 26, 27). A recent study indicated that ISGylation of MDA5 increased downstream signaling and cytokine production (24). Since chronic or repetitive inflammatory signaling can promote malignant transformation (28) and type I interferon signaling can promote cancer stem cell generation (29), an upregulation of ISGylation in FTE cells due to BRCA1 deficiency or in response to follicular fluid exposure could contribute to HGSTOC development. We therefore determined if ISG15 or ISGylation enhances the response to cGAS-STING or MDA5 and RIGI activation in immortalized nonmalignant human FTE cells.

## Results

### Establishing ISG15 and UBA7-null FTE cells

**Figure 1:**
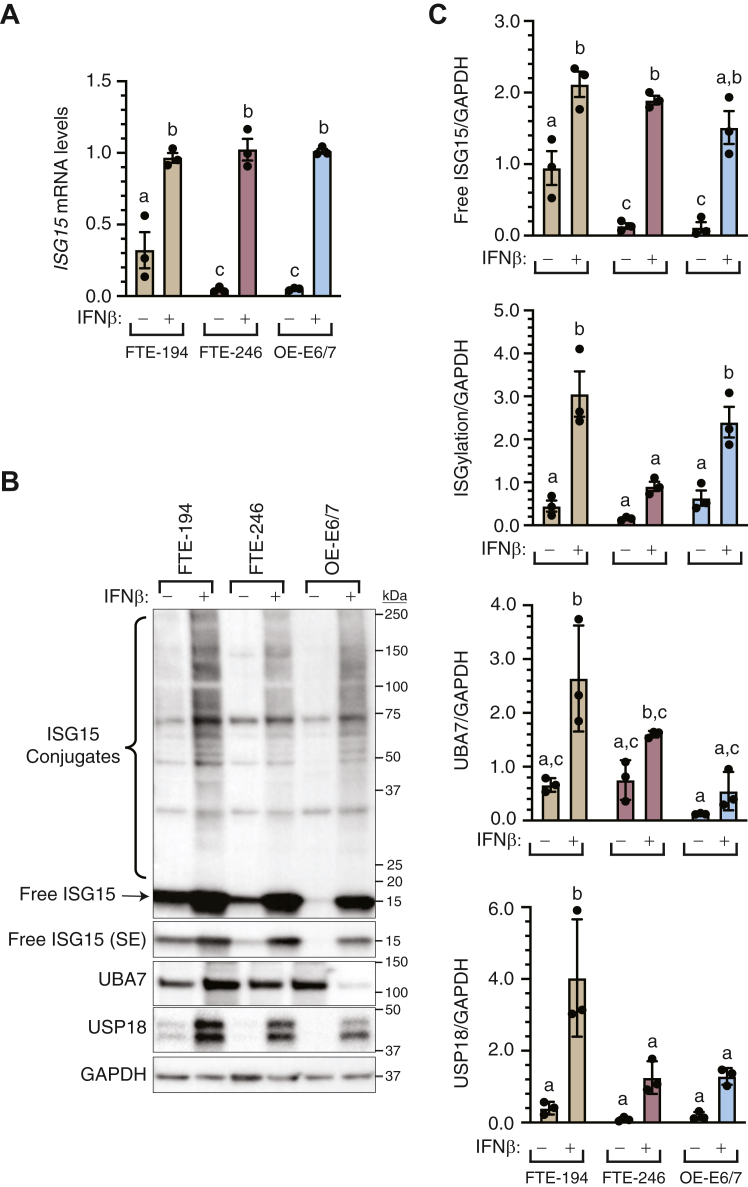
**Screening of FTE cell lines for ISG15, UBA7, and USP18 expression.** FTE-194, FTE-246, and OE-E6/7 cells were treated with 1000 IU/ml IFNβ 24 h prior to harvesting. *A*, relative abundance of *ISG15* transcript levels in cells treated with or without IFNβ as determined by qPCR. *B*, representative Western blot showing levels of overall ISGylated proteins, free ISG15, UBA7, and USP18. GAPDH was used as a loading control for normalization. *C*, bar graphs showing protein levels of free and conjugated ISG15, UBA7, and USP18 in cells treated with or without IFNβ. Bars represent the group mean ± SEM of three independent experiments. Bars with the same letter are not statistically different from one another as determined by a two-way ANOVA, followed by a Holm–Sidak multiple comparison test. FTE, fallopian tube epithelial; IFNβ, interferon β; ISG, interferon-stimulated gene; qPCR, quantitative PCR; SE, short exposure.

Three immortalized human FTE cell lines were screened for expression of *ISG15*, *UBA7*, and *USP18* and capacity for ISGylation. Since these genes are stimulated by interferons, their expression was assessed with or without interferon β (IFNβ) treatment. All three cell lines showed increased *ISG15* transcript abundance (Fig. 1*A*) and free ISG15 protein levels (Fig. 1, *B* and *C*) following IFNβ treatment. FTE-194 cells exhibited higher levels of *ISG15* transcripts and protein levels than FTE-246 and OE-E6/7 cells, although comparable levels were measured in all cell lines following IFNβ treatment. UBA7 and USP18 were detected in all three cell lines and while levels appeared to increase with IFNβ treatment, this achieved statistical significance only in FTE-194 cells. As expected, ISGylation was increased by IFNβ treatment, with highest levels observed in FTE-194 and OE-E6/7 cells. Based on their more robust response to IFNβ, FTE-194 cells were selected to generate sublines devoid of ISG15 (ISG15-null) or lacking capacity for ISGylation (UBA7-null).

**Figure 2:**
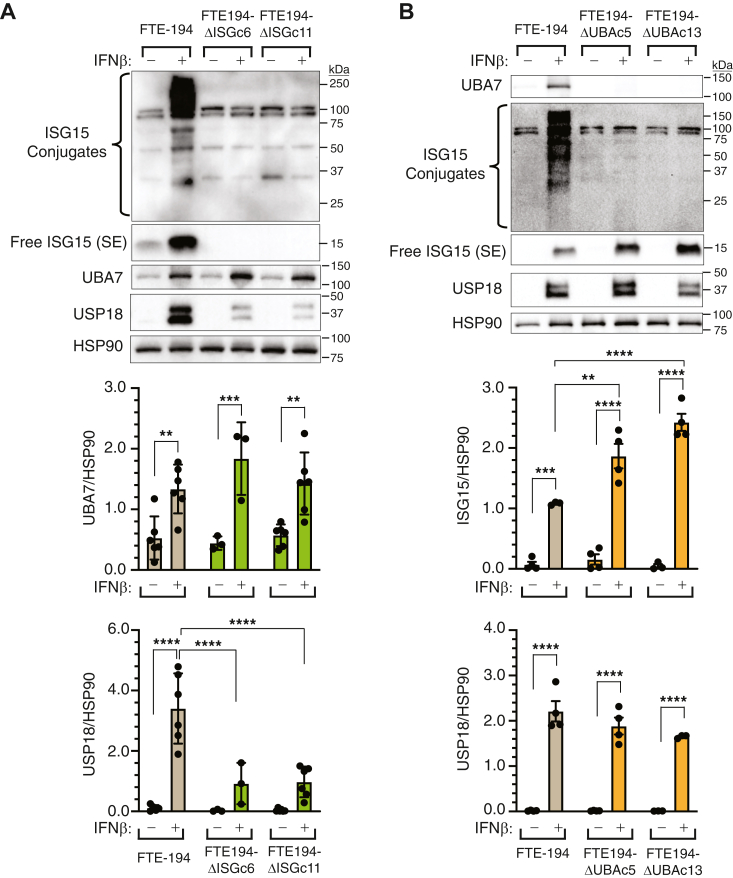
**Loss of ISG15 (ΔISG), but not ISGylation (ΔUBA), decreased USP18 protein levels.** FTE-194 cells and two clonally selected FTE-194 cell lines with disrupted *ISG15* (FTE-194ΔISGc6 cells generated by conventional CRISPR and FTE-194ΔISGc11 cells generated by prime editing) or *UBA7* (FTE-194ΔUBAc5 and c13 cells generated by conventional CRISPR) were treated with or without 1000 IU/ml IFNβ 24 h prior to harvesting. Representative Western blots showing levels of overall ISGylated proteins, free ISG15, UBA7, and USP18. HSP90 was used as a loading control for normalization. *A*, representative Western blot and bar graphs showing levels of UBA7 and USP18 in FTE-194 and FTE-194ΔISG cells treated with or without IFNβ. *B*, representative Western blot and bar graphs showing levels of free ISG15 and USP18 in FTE-194 and FTE-194ΔUBA cells treated with or without IFNβ. Bars represent the group mean ± SEM of 3 to 6 independent experiments. ∗∗*p* ≤ 0.01, ∗∗∗*p* ≤ 0.001, ∗∗∗∗*p* ≤ 0.0001. FTE, fallopian tube epithelial; IFNβ, interferon β; ISG, interferon-stimulated gene; SE, short exposure.

CRISPR gene editing was used to disrupt *ISG15* or *UBA7* in FTE-194 cells to determine the potential impact of both free and conjugated ISG15, and conjugated ISG15 alone, on cGAS-STING and on MDA5 and RIGI signaling. UBA7 protein levels were unaffected by loss of ISG15, with comparable levels of IFNβ-induced UBA7 detected in parental and ISG15-null cells (FTE-194ΔISG) (Fig. 2*A*). Two immunoreactive bands were detected for USP18; a full-length protein of about 39 kDa and an N terminus–deleted isoform of about 34 kDa (30). Loss of ISG15 resulted in reduced levels of USP18 (Fig. 2*A*); however, this was expected since free ISG15 is known to stabilize USP18 (18). Loss of UBA7 (FTE-194ΔUBA) resulted in the absence of conjugated ISG15 and an increase in free ISG15 levels induced by IFNβ treatment (Fig. 2*B*). Neither baseline nor IFNβ-induced levels of USP18 were affected by loss of UBA7 (Fig. 2*B*).

### Effect of ISG15 or UBA7 deletion on cytosolic dsDNA signaling

**Figure 3:**
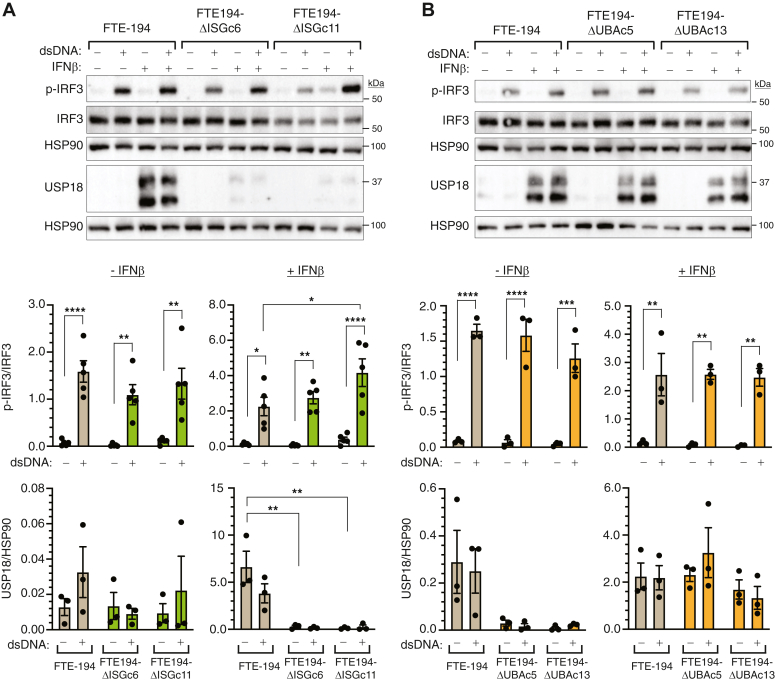
**Loss of ISGylation does not impact activation of IRF3 by dsDNA90.** Representative Western blots and bar graphs summarizing phosphorylated IRF3 (p-IRF3) levels in parental FTE-194 cells and two clonally selected ISG15-null cell sublines (*panel A*) or in parental FTE-194 cells and two clonally selected UBA7-null cell sublines (*panel B*). Cells pretreated with or without 500 IU/ml IFNβ were transfected with 0.3 μg dsDNA90 3 h before harvesting. Representative Western blots of p-IRF3, total IRF3, and USP18 are shown with bar graphs summarizing the results of 3 to 5 independent experiments. HSP90 was used as a loading control. Bars represent the group mean ± SEM. ∗*p* ≤ 0.05, ∗∗*p* ≤ 0.01, ∗∗∗*p* ≤ 0.001, and ∗∗∗∗*p* ≤ 0.0001. FTE, fallopian tube epithelial; IFNβ, interferon β; ISG, interferon-stimulated gene.

To determine if loss of total ISG15 or ISGylation alone impacts cGAS-STING signaling in FTE cells, FTE-194 cell lines were transfected with dsDNA90. Preliminary studies with parental cells determined an optimal dose and treatment time (Fig. S1*A*). Experiments were performed in the absence or presence of IFNβ pretreatment in order to test the effect of dsDNA90 treatment under conditions of prior upregulated expression of genes involved in ISGylation. Transfection with dsDNA90 led to activation of IRF3 in both parental and ISG15-null cells in the presence or absence of IFNβ pretreatment (Fig. 3*A*), with higher levels of p-IRF3 measured in IFNβ-pretreated cells. A significant increase in IRF3 activation by dsDNA90 was found in FTE-194ΔISGc11 cells but not in FTE-194ΔISGc6 cells as compared to parental cells and was only observed in cells pretreated with IFNβ (Fig. 3*A*). Loss of capacity for ISGylation did not alter the ability of dsDNA90 to activate IRF3 in either the presence or absence of IFNβ pretreatment in either clonal subline (Fig. 3*B*). Pretreatment with IFNβ led to increased levels of IRF3 activation by dsDNA90 treatment in all cell sublines.

IFNβ pretreatment but not dsDNA90 increased USP18 levels in parental FTE-194 cells and UBA7-null cells, but not in ISG15-null cells (Fig. 3), likely reflecting the importance of free ISG15 in stabilizing USP18. Altogether, these experiments indicate that neither ISGylation nor free ISG15 impact cGAS-STING function in these cells.

### Effect of ISG15 or UBA7 deletion on cytosolic dsRNA signaling

**Figure 4:**
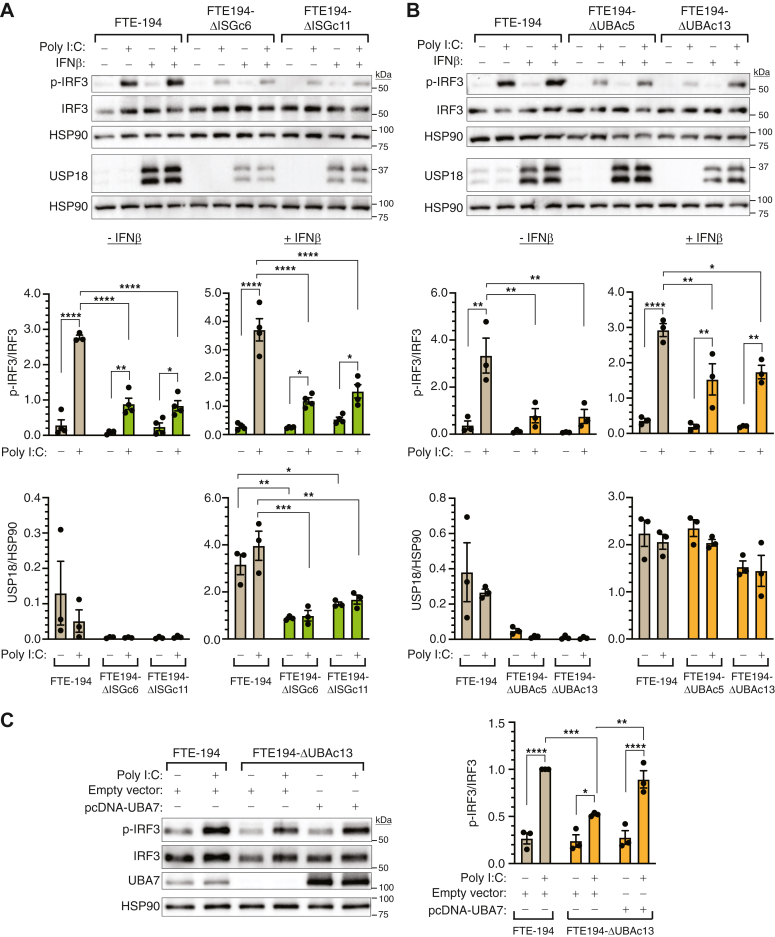
**Loss of ISGylation decreases activation of IRF3 by Poly I:C transfection.***A* and *B*, representative Western blots and bar graphs summarizing p-IRF3, total IRF3, and USP18 levels in parental FTE-194 cells and two clonally selected ISG15-null cell sublines (*panel A*) or in parental FTE-194 cells and two clonally selected UBA7-null cell sublines (*panel B*). Cells pretreated with or without 500 IU/ml IFNβ were transfected with 0.3 μg Poly I:C 1 h before harvesting. *C*, representative Western blot and bar graphs summarizing p-IRF3 and total IRF3 levels in parental FTE-194 cells and UBA7-null cells transfected with pcDNA-UBA7 cDNA or empty vector. Cells were transfected with or without 0.3 μg Poly I:C 1 h before harvesting. Bar graphs summarize the results of 3 to 4 independent experiments. HSP90 was used as a loading control. Bars represent the group mean ± SEM. ∗*p* ≤ 0.05, ∗∗*p* ≤ 0.01, ∗∗∗*p* ≤ 0.001, and ∗∗∗∗*p* ≤ 0.0001. FTE, fallopian tube epithelial; IFNβ, interferon β; ISG, interferon-stimulated gene; Poly I:C, polyinosinic:polycytidylic acid.

We next determined if loss of total ISG15 or ISGylation impacts MDA5 or RIGI signaling. FTE-194 cell lines were treated with polyinosinic:polycytidylic acid (Poly I:C), which functions as a synthetic dsRNA able to stimulate MDA5, RIGI, and TLR3. Cells were transfected with Poly I:C to gain access to intracellularly localized MDA5 and RIGI. Preliminary studies with parental FTE-194 cells determined an optimal dose and treatment time (Fig. S1*B*). In contrast to cGAS-STING, loss of either total ISG15 or the capacity for ISGylation resulted in a 50 to 60% decreased level of p-IRF3 (Fig. 4, *A* and *B*). This effect was observed in both the presence and absence of IFNβ pretreatment, with slightly higher overall levels of p-IRF3 observed with IFNβ pretreatment. A statistically significant decrease in p-IRF3 levels in both ISG15-null clones relative to FTE-194 parental cells was observed (Fig. 4*A*). Similar results were obtained with UBA7-null cells with lower p-IRF3 levels observed in both UBA7-null clones relative to FTE-194 parental cells (Fig. 4*B*).

As expected, IFNβ pretreatment but not Poly I:C treatment increased USP18 protein levels at this time point. Consistent with the role of free ISG15 in stabilizing USP18, levels of IFNβ-induced USP18 were lower in ISG15-null cells but not in UBA7-null cells as compared to parental control cells (Fig. 4, *A* and *B*).

To verify that decreased activation of IRF3 by intracellular Poly I:C was due to the loss of ISGylation capacity, we examined if exogenous UBA7 expression in UBA7-null FTE-194 cells reversed the effect. UBA7 overexpression in UBA7-null cells increased p-IRF3 levels following Poly I:C transfection to levels 2-fold higher than that measured in UBA7-null cells transfected with empty expression vector and similar to that measured in parental FTE-194 cells (Fig. 4*C*).

**Figure 5:**
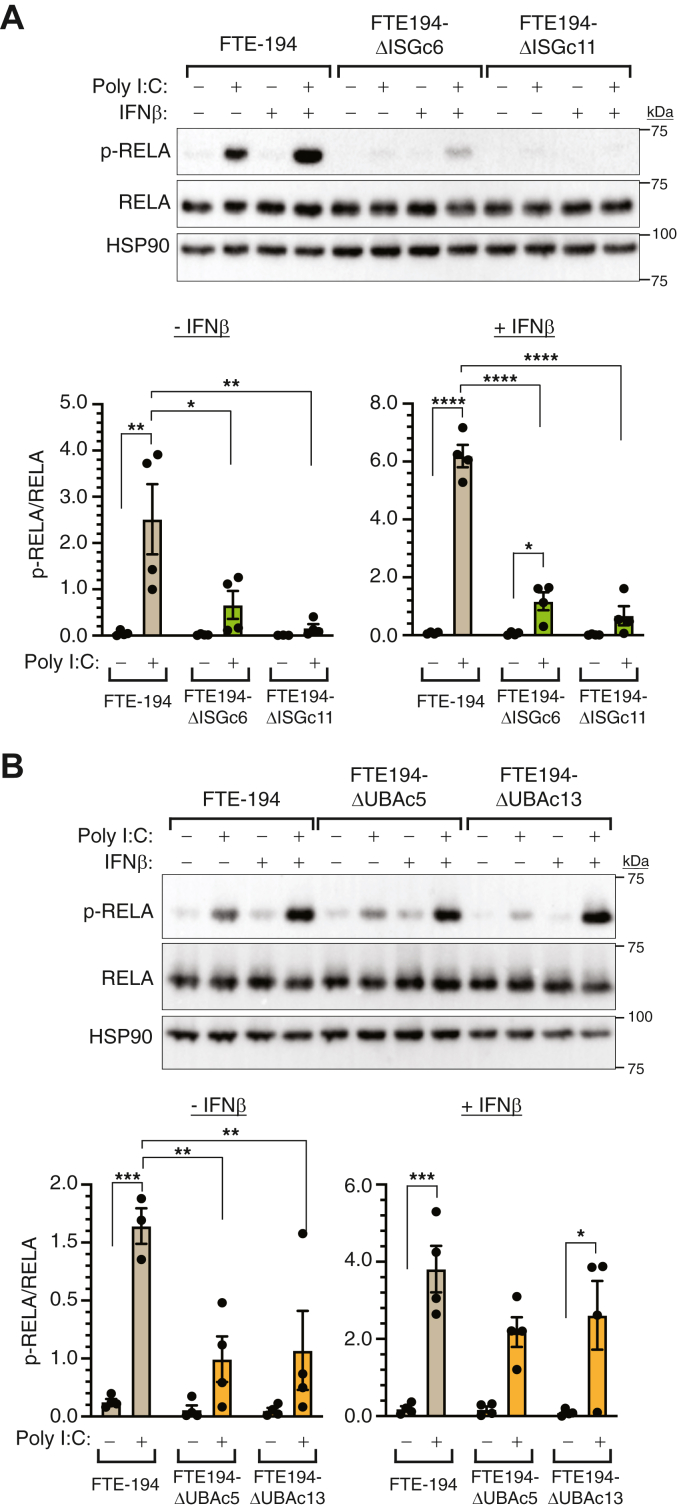
**Loss of ISGylation decreases activation of NFκB by Poly I:C transfection.** Representative Western blots and bar graphs summarizing p-RELA levels in parental FTE-194 cells and two clonally selected ISG15-null cell sublines (*panel A*) or in parental FTE-194 cells and two clonally selected UBA7-null cell sublines (*panel B*). Cells pretreated with or without 500 IU/ml IFNβ were transfected with 0.3 μg Poly I:C 1 h before harvesting. Representative Western blots of p-RELA and total RELA are shown with bar graphs summarizing the results of 3 to 4 independent experiments. HSP90 was used as a loading control. Bars represent the group mean ± SEM. ∗*p* ≤ 0.05, ∗∗*p* ≤ 0.01, ∗∗∗*p* ≤ 0.001, ∗∗∗∗*p* ≤ 0.0001. FTE, fallopian tube epithelial; IFNβ, interferon β; ISG, interferon-stimulated gene; Poly I:C, polyinosinic:polycytidylic acid.

Stimulation of RIGI or MDA5 activates NFκB signaling in addition to IRF3; thus, we anticipated that loss of total ISG15 or ISGylation would also lead to decreased RELA/p65 activation by Poly I:C. Similar to IRF3, activation of RELA by Poly I:C transfection was diminished in ISG15-null and UBA7-null cells (Fig. 5). Overall Poly I:C–induced levels of p-RELA were increased in cells pretreated with IFNβ, which was expected due to an increase of interferon stimulated genes including MDA5 and RIGI. Poly I:C transfection led to an increase in p-RELA levels in parental cells in both the presence or absence of IFNβ pretreatment, whereas only FTE-194ΔISG15 clone 6 cells pretreated with IFNβ showed a statistically significant increase in p-RELA levels by Poly I:C treatment. Nevertheless, the levels of p-RELA induced by Poly I:C in ISG15-null cells was 25% or less than that induced in parental cells (Fig. 5*A*).

Loss of UBA7 also decreased the impact of Poly I:C transfection on RELA activation; however, this was only observed in the absence of IFNβ pretreatment (Fig. 5*B*). In the absence of IFNβ pretreatment, the level of p-RELA in UBA7-null cells induced by Poly I:C was approximately 30% that induced in parental cells (Fig. 5*B*). Higher levels of Poly I:C–induced p-RELA were measured in cells pretreated with IFNβ with no statistical difference between parental and UBA7-null cells.

### Effect of ISG15 or UBA7 on intracellular dsRNA-mediated gene expression

**Figure 6:**
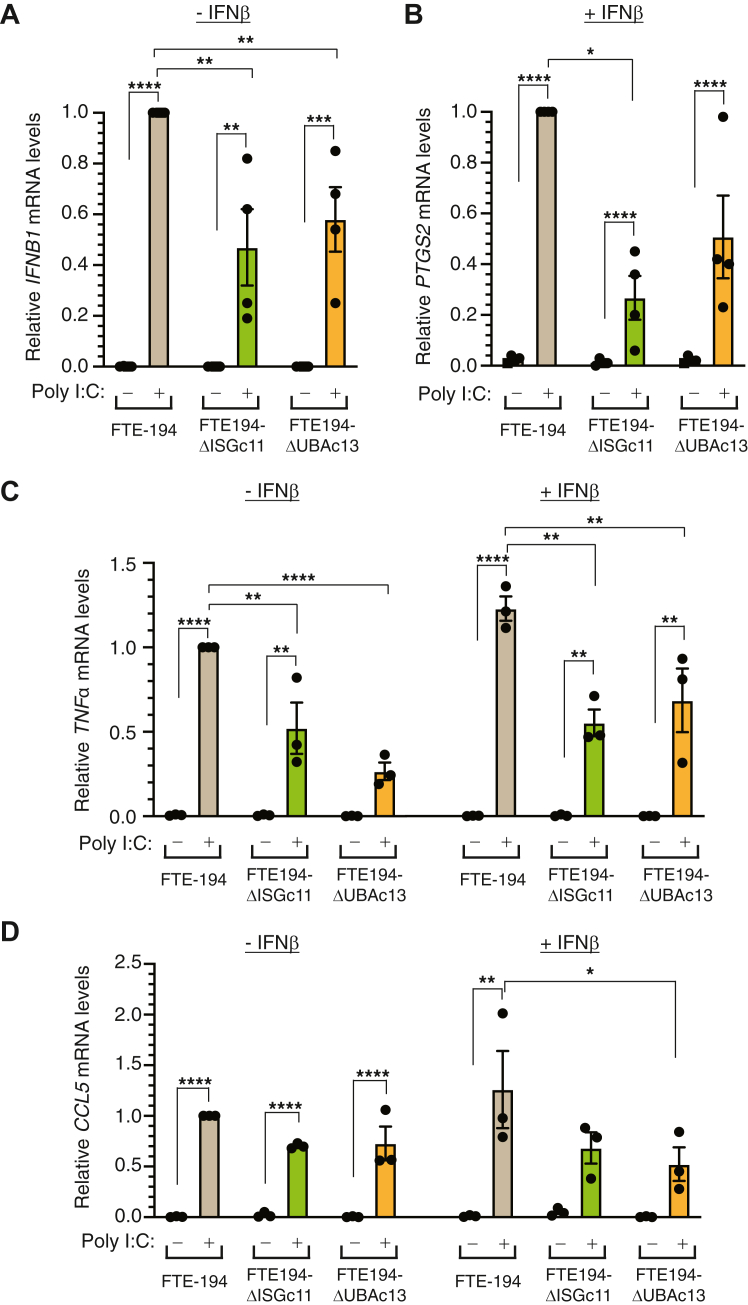
**Loss of ISG15 or UBA7 decreases activation of downstream targets of IRF3 and NFκB.***A* and *B*, relative *IFNB1* (*panel A*) and *PTGS2* (*panel B*) transcript levels measured by qPCR in parental, ISG15-null, and UBA7-null FTE-194 cells transfected with 0.3 μg Poly I:C for 1.5 h. Values are relative to levels measured in parental cells treated with Poly I:C. *C*, relative *TNFα* transcript levels measured in cells transfected with 0.3 μg Poly I:C for 3 h. Cells were treated with or without 500 IU/ml IFNβ. Values are relative to levels measured in parental cells treated with Poly I:C in the absence of IFNβ pretreatment. *D*, relative *CCL5* transcript levels measured in cells transfected with 0.3 μg Poly I:C for 8 h. Cells were treated with or without 500 IU/ml IFNβ. Values are relative to levels measured in parental cells treated with Poly I:C in the absence of IFNβ pretreatment. Bars represent the group mean ± SEM of 3 to 4 independent experiments. ∗*p* ≤ 0.05, ∗∗*p* ≤ 0.01, ∗∗∗*p* ≤ 0.001, and ∗∗∗∗*p* ≤ 0.0001. FTE, fallopian tube epithelial; IFNβ, interferon β; ISG, interferon-stimulated gene; Poly I:C, polyinosinic:polycytidylic acid; qPCR, quantitative PCR.

IRF3 activation induces type I interferon production. To determine if the diminished activation of IRF3 in ISG15-null and UBA7-null cells is reflected in decreased IFNβ expression, FTE-194, ΔISG15c11, and ΔUBA7c13 cells were transfected with Poly I:C and transcript levels of *IFNB1* were assessed by quantitative PCR (qPCR). Poly I:C transfection increased *IFNB1* transcript levels in all three cell sublines, with levels induced in ISG15-null and UBA7-null cells approximately 50% that induced in parental cells (Fig. 6*A*).

*PTGS2/COX2* and *TNFα* are direct targets of NFκB signaling. To determine if the diminished activation of RELA in ISG15-null and UBA7-null cells is reflected in decreased activation of either *PTGS2* or *TNFα* expression, FTE-194, ΔISG15c11, and ΔUBA7c13 cells were transfected with Poly I:C and transcript levels assessed by qPCR. Poly I:C transfection led to increased *PTGS2* transcript levels in all three cell lines that was reduced in ISG15-null and UBA7-null cells to 30 to 50% of that in parental cells (Fig. 6*B*). The impact of Poly I:C transfection on *TNFα* transcript levels was determined in the presence and absence of IFNβ pretreatment. Poly I:C transfection increased *TNFα* transcript levels with a 50 to 70% reduction in Poly I:C–induced levels measured in ISG15-null and UBA7-null cells as compared to parental cells (Fig. 6*C*). Similar results were obtained in the presence or absence of IFNβ pretreatment.

CCL5/RANTES is a chemokine activated by Poly I:C through type I interferons and NFκB activation (31, 32). *CCL5* transcript levels were increased by Poly I:C transfection but not by IFNβ pretreatment (Fig. 6*D*). Lower levels of Poly I:C-induced *CCL5* transcripts were measured in ISG15-null and UBA7-null cells; however, multiple comparison testing indicated that this reached statistical significance only for UBA7-null cells pretreated with IFNβ (Fig. 6*D*).

### Effect of ISG15 and UBA7 deletion on extracellular dsRNA sensing

In contrast to MDA5 and RIGI, TLR3 senses dsRNA in endosomes and can activate IRF3 and NFκB signaling (33) when Poly I:C is added directly to culture medium without lipofectamine (34). Preliminary studies determined an optimal dose and treatment time for Poly I:C to stimulate IRF3 (Fig. S1, *C* and *D*). The addition of naked Poly I:C to culture medium resulted in the activation of IRF3 in ISG15-null (Fig. S2*A*) and UBA7-null (Fig. S2*B*) cells that was similar to that of parental FTE-194 cells, with levels unaffected by IFNβ pretreatment. Similarly, loss of ISG15 or UBA7 did not alter the ability of Poly I:C added to the culture medium to activate RELA. USP18 levels were detectable only in cells pretreated with IFNβ and were unaffected by naked Poly I:C treatment (Fig. S2, *C* and *D*). Lower levels of IFNβ-induced USP18 levels were observed in ISG15-null cells (Fig. S2*C*) and in clone 13 UBA7-null cells than in parental cells (Fig. S2*D*).

### RIGI mediation of intracellular Poly I:C effects

**Figure 7:**
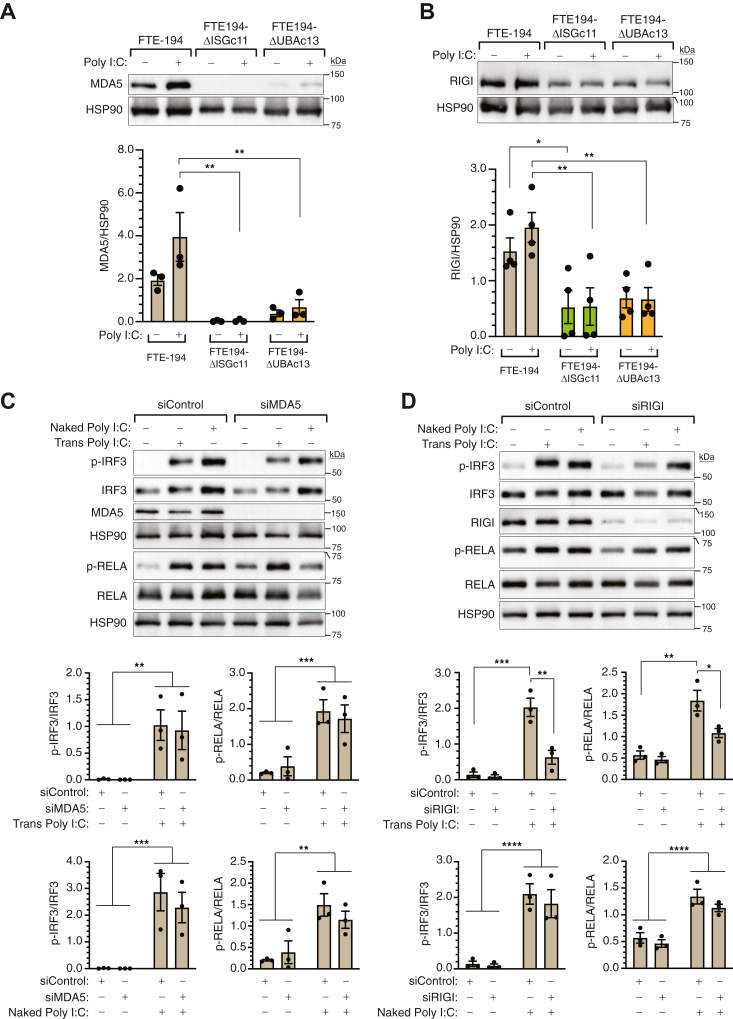
**Loss of ISGylation decreases both MDA5 and RIGI levels and RIGI mediates transfected Poly I:C effects.***A* and *B*, representative Western blots and bar graph summarizing MDA5 (*panel A*) or RIGI levels (*panel B*) in cells transfected with or without 0.3 μg Poly I:C for 1 h. Two-way ANOVA indicated an effect of ISG15 and MDA5 status (*p* = 0.0002) and for ISG15 and RIGI status (*p* = 0.0003). *C* and *D*, representative Western blots and bar graph summarizing p-IRF3 and p-RELA levels in parental FTE-194 cells transfected with nontargeting siRNA or siRNA targeting either MDA5 (*panel C*) or RIGI (*panel D*) 72 h prior to transfection of 0.3 μg Poly I:C (Trans Poly I:C) for 1 h or addition of 0.3 μg naked Poly I:C for 2 h. *Panel C:* two-way ANOVA indicated a significant effect of Poly I:C transfection or addition on both p-IRF3 and p-RELA levels (*p* < 0.005), but no effect of siMDA5 or interaction. *Panel D:* two-way ANOVA indicated a significant effect of Poly I:C and siRIGI transfection on both p-IRF3 and p-RELA levels (*p* < 0.02) and a significant interaction between Poly I:C and siRIGI on p-IRF3 levels (*p* = 0.0037). For naked Poly I:C treatment, two-way ANOVA indicated a significant effect of Poly I:C treatment on both p-IRF3 and p-RELA levels (*p* < 0.0001), but no effect of siRIGI or interaction. Bars represent the group mean ± SEM of 3 to 4 independent experiments. Significant group comparisons as determined by Holm–Sidak’s multiple comparison testing are shown. ∗*p* ≤ 0.05, ∗∗*p* ≤ 0.01, ∗∗∗*p* ≤ 0.001, and ∗∗∗∗*p* ≤ 0.0001. FTE, fallopian tube epithelial; ISG, interferon-stimulated gene; Poly I:C, polyinosinic:polycytidylic acid.

Both MDA5 and RIGI are expressed by FTE-194 cells and are demonstrated targets of ISGylation (24, 25). Loss of ISG15 or UBA7 in FTE-194 cells resulted in a marked decrease in both MDA5 and RIGI (Fig. 7, *A* and *B*). To determine which of these dsRNA sensors mediate the effects of transfected Poly I:C on IRF3 and RELA activation in FTE-194 cells, targeting siRNA was used to downregulate MDA5 or RIGI levels prior to Poly I:C treatment. Preliminary studies determined the optimal dose and duration of siRNA treatment in the presence and absence of IFNβ treatment (Fig. S3). FTE-194 cells were transfected with nontargeting siRNA or siRNA targeting MDA5 or RIGI 72 h prior to transfection or addition of Poly I:C. Cells were harvested after Poly I:C transfection or addition of naked Poly I:C and levels of p-IRF3 and p-RELA were determined by Western blot analysis. Knockdown of MDA5 levels did not impact activation of IRF3 or RELA induced by transfected Poly I:C (Fig. 7*C*). However, knockdown of RIGI decreased p-IRF3 levels following transfection of Poly I:C by 65% and levels of p-RELA by 40% (Fig. 7*D*). As expected, neither knockdown of MDA5 nor RIGI affected activation of IRF3 or RELA by addition of naked Poly I:C to the culture medium (Fig. 7, *C* and *D*).

### Impact of loss of ISG15 or UBA7 on cell growth and migration

**Figure 8:**
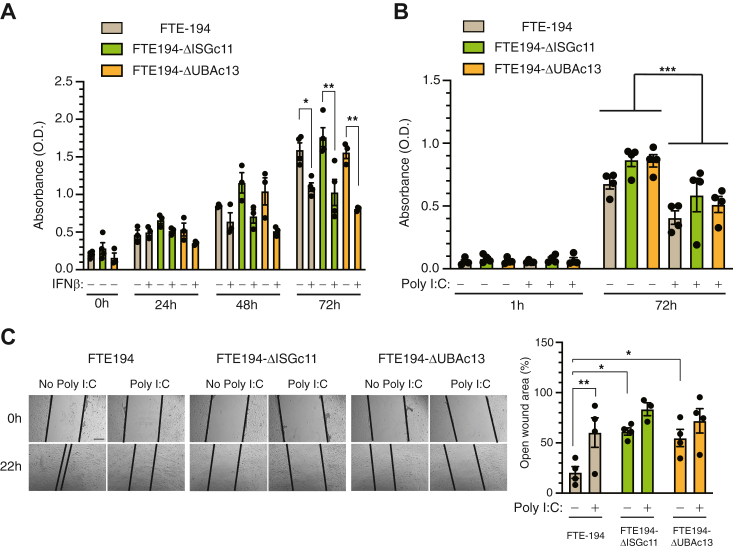
**Loss of ISG15 or ISGylation did not impact cell culture growth but decreased cell migration.***A*, cells were treated with IFNβ (500 IU/ml) or vehicle 24 h after seeding. Absorbance, reflecting relative cell number, was determined 0, 24, 48, and/or 72 h later using an XTT dye reduction assay. Two-way ANOVA performed on 72 h data indicated an effect of IFNβ treatment (*p* < 0.0001) only. Individual group comparisons by a Holm–Sidak multiple comparison test are shown. *B*, cells were transfected with or without Poly I:C (0.3 μg) for 1 h and seeded into 96-well plates. Absorbance was determined 72 h later. Two-way ANOVA indicated an effect of Poly I:C treatment (*p* = 0.0054) only. Individual group comparisons by a Holm–Sidak multiple comparison test are shown. *C*, representative images of scratch-wound healing in parental, ISG15-null, and UBA7-null FTE-194 cells treated with 0.3 μg Poly I:C for 1 h and bar graph summarizing the amount of open wound remaining at 22 h from 3 to 4 independent experiments, each with 2 to 3 replicates. The scale bar represents 300 μm. ∗*p* ≤ 0.05, ∗∗*p* ≤ 0.01, and ∗∗∗*p* ≤ 0.001. FTE, fallopian tube epithelial; IFNβ, interferon β; ISG, interferon-stimulated gene; Poly I:C, polyinosinic:polycytidylic acid.

Type I interferons exert both protumorigenic and antitumorigenic effects in ovarian cancer, including inhibition of cell proliferation and migration (35). An XTT dye-reduction assay was used to assess the impact of loss of ISG15 or UBA7 on cell culture growth following treatment with IFNβ or intracellular Poly I:C. Loss of ISG15 or UBA7 alone did not affect the growth of the cell cultures. While both IFNβ and Poly I:C decreased culture growth, the loss of either ISG15 or UBA7 had no effect on this decrease (Fig. 8, *A* and *B*).

Cell migration was assessed using a scratch-wound assay. Loss of ISG15 or UBA7 slowed cell migration as compared to parental cells (Fig. 8*C*). Poly I:C treatment slowed migration in parental cells to that observed in ISG15-null and UBA7-null cells; however, Poly I:C had no further effect on migration of either ISG15-null or UBA7-null cells, indicating that ISGylation promotes cell migration but is required for the inhibitory effect of dsRNA.

### Impact of loss of ISG15 or UBA7 on spheroid formation and stem gene transcript levels

**Figure 9:**
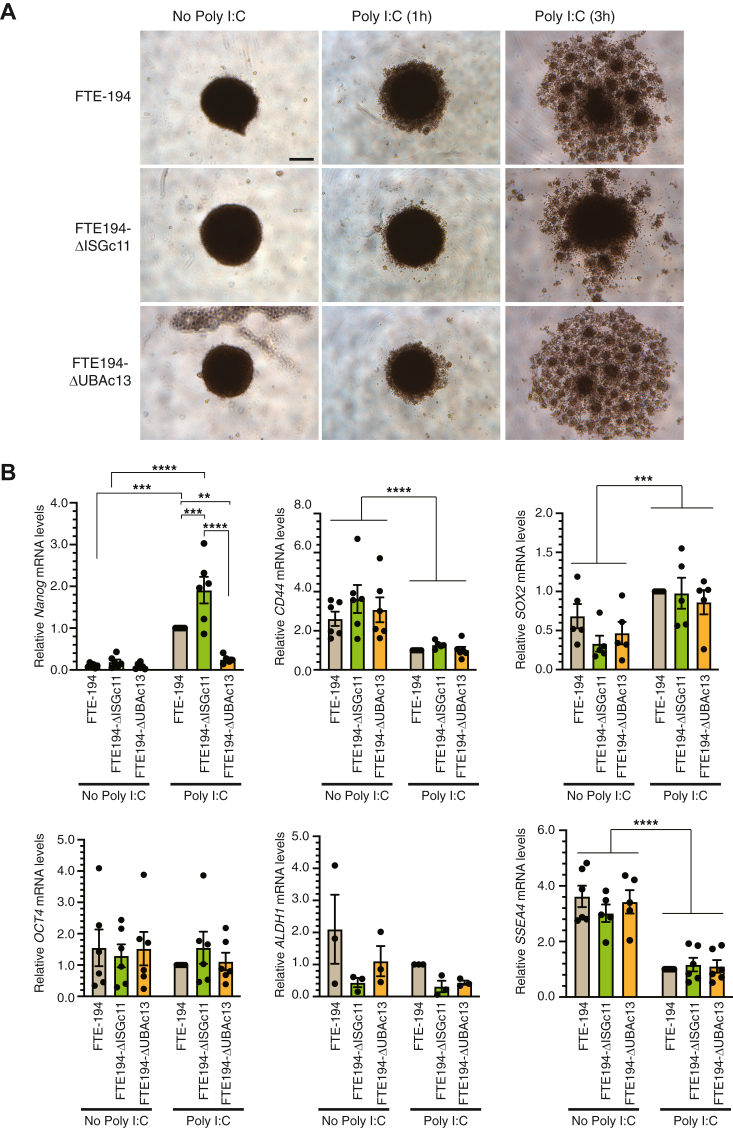
**Impact of loss of ISG15 or UBA7 on spheroid formation and transcript levels of stemness genes.***A*, representative images of spheroids formed by parental, ISG15-null, and UBA7-null FTE-194 cells transfected with or without 0.3 μg Poly I:C for 1 or 3 h. Identical results were obtained in three independent experiments, each with 2 to 3 replicates. The scale bar represents 300 μm. *B*, relative transcript levels of *NANOG*, *CD44*, *SOX2*, *OCT4*, *ALDH1*, and *SSEA4* measured by qPCR in parental, ISG15-null, and UBA7-null FTE-194 cells 3 h after transfection with or without 0.3 μg Poly I:C. Values are relative to levels measured in parental cells treated with Poly I:C. Bars represent the group mean ± SEM of 3 to 6 independent experiments. Significant group comparisons as determined by ANOVA followed by Holm–Sidak’s multiple comparison testing are shown. ∗∗*p* ≤ 0.01, ∗∗∗*p* ≤ 0.001, and ∗∗∗∗*p* ≤ 0.0001. FTE, fallopian tube epithelial; ISG, interferon-stimulated gene; Poly I:C, polyinosinic:polycytidylic acid; qPCR, quantitative PCR.

Wang *et al.* (36) reported a MAVS-dependent increase in expression of stemness genes, including *NANOG* and *OCT3/4*, in human fibroblasts following transfection with Poly I:C. Moreover, type I interferons have recently been implicated in the generation of cancer stem cells (29). FTE-194 cells exhibit an inherent capacity to form tight spheroids (37), which serves as an indicator of stemness. Consistent with this property, parental FTE-194 cells express *SOX2*, *CD44*, *OCT3/4*, *SSEA4*, and *ALDH1*, but few *NANOG* transcripts (Fig. 9). The effect of loss of ISG15 or UBA7 and intracellular Poly I:C treatment on the spheroid forming ability of FTE-194 cells and on transcript levels for stemness genes was examined. Loss of ISG15 or UBA7 alone did not appreciably alter spheroid formation with the exception that spheroids were slightly larger in ISG15 null cells (Fig. 9*A*). Intracellular Poly I:C prevented tight spheroid formation in parental cells and in UBA7-null cells, but not in ISG15-null cells. This retention of tight spheroid formation capacity in ISG15-null cells occurred despite marked reductions in *CD44*, *OCT3/4*, and *SSEA4* transcript levels, which also occurred in parental and UBA7-null cells (Fig. 9). Intracellular Poly I:C treatment increased the levels of *NANOG* transcripts 10-fold in parental cells, with levels further increased in ISG15-null cells (Fig. 9*B*). In contrast, Poly I:C treatment failed to increase *NANOG* transcript levels in the absence of ISGylation. The finding of retention of tight spheroid formation and increased *NANOG* transcript levels in ISG15-null cells was further demonstrated in another clonal ISG15-null cell line (Fig. S4). These results indicate that *NANOG* expression is suppressed by free ISG15 and increased through ISGylation.

## Discussion

Activation of the innate immune response is often considered antagonistic to tumor formation and progression, partly due to the growth inhibitory actions of type I interferons and their priming activity for maturation of antigen presenting cells. Stimulation of pattern recognition receptors, the keystones of the innate immune system, triggers release of proinflammatory cytokines, largely through activation of NFκB, which can promote carcinogenesis (38). Unresolved or prolonged proinflammatory cytokine signaling can result in increased proliferation and accumulation of genetic mutations and epigenetic changes largely mediated by overproduction of reactive oxygen and nitrogen species (reviewed by (28)). Moreover, while interferons can reduce tumor cell growth and survival, recent evidence indicates that type I interferons can promote reprograming of cancer cells to generate chemotherapy and immunetherapy-resistant cancer stem cells (29).

In the present study, we demonstrate a key role of ISGylation in enhancing proinflammatory signaling stimulated by intracellular dsRNA in immortalized nonmalignant FTE cells. Deletion of total ISG15, through genomic disruption of *ISG15*, or the capacity for ISGylation, through disruption of *UBA7*, suppressed activation of both NFκB and IRF3 by Poly I:C, resulting in decreased transcript levels of downstream inflammatory target genes. As a dsRNA mimetic, Poly I:C can stimulate MDA5, RIGI, and TLR3. Both MDA5 and RIGI are localized to the cytoplasm, whereas TLR3 largely resides within endosomes and thus detects endocytosed extracellular dsRNA. Naked Poly I:C is taken up by cells through endosomes, and thus stimulates TLR3 signaling to activate IRF3 and NFκB. Our data indicate that neither free ISG15 nor ISGylation impacted TLR3 sensitivity or signaling in these cells since loss of either ISG15 or UBA7 did not alter IRF3 or RELA activation when naked Poly I:C was added to the culture medium. Moreover, this finding, together with our finding that loss of ISG15 or UBA7 did not affect dsDNA90 sensing, indicated that the protein target of ISGylation that enhances dsRNA sensing lies upstream of IRF3 and NFκB.

Our results indicate that the effects of Poly I:C in FTE-194 cells are mediated primarily through RIGI activation. Nevertheless, a marked decrease in MDA5 and RIGI was observed in ISG15-null and UBA7-null cells, indicating that dsRNA sensing by both sensors is likely to be impacted. ISGylation of both MDA5 and RIGI has been reported. ISGylation of MDA5 results in stabilization of its activated state (24), whereas RIGI ISGylation resulted in the decreased stability of nonconjugated RIGI and decreased downstream signaling in hepatocellular cancer cells (25). The results of the present study indicate that expression of ISG15 and ISGylation in FTE-194 cells acts to stabilize RIGI as well as MDA5. RIGI protein levels are regulated by multiple signaling pathways controlling posttranscriptional modification, particularly ubiquitination. RIGI is degraded by proteasomes following its ubiquitination by the E3 ligase RNF125 (39) or by autophagosomes (40). ISGylation of RIGI could directly prevent RNF125 from interacting with RIGI, thereby stabilizing protein levels, or could act indirectly to impact the availability of RNF125 or autophagosomes (41). We were unable to demonstrate endogenous ISGylation of RIGI in FTE-194 cells; thus, it remains possible that the effect of ISGylation on RIGI stability and signaling could be mediated by other targeted proteins. However, only a fraction (5–10%) of target protein is typically ISGylated (42), making it difficult to demonstrate ISGylation of endogenous target proteins that are not expressed in abundance. In fact, the majority of studies identifying targets of ISGylation to date, including MDA5 and RIGI, have been performed in cells overexpressing tagged ISG15 (43).

ISGylation potentially controls the stability, activity, and subcellular localization of hundreds of client proteins (44, 45). The overall effects of ISGylation in cancer are thus expected to be complex and context-dependent. *ISG15* expression is increased in several epithelial cancers—including bladder, breast, and prostate (46, 47, 48, 49)—and is increased in relapsed HGSTOC tumors as compared to primary tumors (50). ISG15 contributes to rat sarcoma virus protein-induced oncogenic transformation of breast epithelial cells (51, 52) and disrupts F-actin architecture and focal adhesion formation to promote migration of breast cancer cells (53). Induction of ISGylation in mice exacerbates intestinal inflammation, increases ROS, and promotes generation of colitis-associated colon cancer (54).

ISG15 and USP18 exert effects independent of ISGylation. While free ISG15 is found intracellularly, it is also secreted and acts upon lymphocytes to induce release of IFNγ and possibly other cytokines (55). Secreted ISG15 suppressed tumor growth, increased natural killer cell tumor infiltration, and enhanced cell surface MHC class I expression in breast tumors (56). A primary role of intracellular unconjugated ISG15 in humans, but not in mice, is to stabilize USP18, which leads to the inhibition of the interferon response (57). Thus, free ISG15 may have differential and beneficial effects on cancer progression as compared to ISGylation, raising the possibility that selectively targeting ISGylation, while preserving or enhancing free ISG15 levels, may be of preventative value. Elevated interferon signaling has been observed in various *BRCA1*-null or mutated cancers (58, 59, 60) and epithelial cells (61). Increased expression of *ISG15* mRNA was detected in a triple-negative *BRCA1*-null breast cancer cell line compared to cells transfected with *BRCA1*. As well, elevated interferon signaling, including ISG15 levels, was observed in an ovarian cancer *BRCA1*-null cell line compared to *BRCA1*-transfected cells (62).

BRCA1 deficiency imparts a strong predilection for malignant transformation by FTE cells. BRCA1 is most notably involved in initiating homologous recombination at DNA double-strand breaks to enact high-fidelity repair and to prevent nucleolytic degradation of nascent DNA strands at stalled replication forks. Loss of BRCA1 results in accumulation of DNA base substitutions, insertion or deletion of base sequences, and chromosomal rearrangements that typically trigger cell cycle arrest and death; however, when combined with loss of TP53 activity, these damaged cells survive and can give rise to tumors. Mitotic mis-segregation of chromosomal fragments into micronuclei that accumulate and rupture in BRCA1-deficient cells can give rise to cytosolic dsDNA that stimulates cGAS-STING and inflammatory signaling, which may drive cancer initiation. BRCA1 deficiency also results in an increase in RNA polymerase III that is capable of generating dsRNA from the accumulated DNA, thereby activating MDA5 and RIGI (63, 64). Furthermore, loss of BRCA1 in human ovarian cancer cells suppresses nuclear R-loop formation, resulting in accumulation of RNA-DNA hybrids in the cytoplasm (65) which have the potential to activate RIGI (64).

Activation of cGAS-STING has been reported in breast epithelial cells from *BRCA1* mutation carriers and in BRCA1-deficient HGSTOC, which was associated with an increased inflammatory state (19). BRCA1-deficient ovarian cancer cells exhibited increased transcript levels of *MDA5* and *RIGI* as well as multiple downstream ISGs including *IRF3* and *IRF7*. Interestingly, this finding corroborated our previous work showing increased transcript levels of *MDA5* and *RIGI* in nonmalignant primary FTE cells from *BRCA1*-mutation carriers, as compared to FTE cells from control patients, following their exposure to periovulatory follicular fluid (13). Further highlighting the potential importance of RIGI and MDA5 in ovarian cancer, de Queiroz (66) found that cGAS-STING activation, but not that of MDA5 or RIGI, was impaired in a majority of ovarian cancer cell lines.

In 2012, Lee *et al.* (67) serendipitously found that activation of the innate immune response during retroviral transfection of induced pluripotency genes enhanced the reprogramming of target fibroblast cells. They reported that activation of TLR3 by Poly I:C treatment downregulated histone deacetylases, thereby facilitating chromatin remodeling. More recently, Wang *et al.* (36) found that transfection of Poly I:C into human fibroblast cells, but not addition of naked Poly I:C, increased *OCT3/4* and *NANOG* expression as well as other stemness genes. This induction was found to be due to MAVS-dependent activation of IRF1, consistent with RIGI or MDA5 activation. Qadir *et al.* (68, 69) found that type I interferon production resulting from chronic activation of CD95/FAS led to an increased number of stem cells through STAT1 activation in multiple breast cancer cell lines. Interestingly, Alcala *et al.* (70) found that ISG15 produced by pancreatic cancer stem cells enhanced their ability to self-renew and generate tumors *in vivo*. Similarly, ISG15 expression promoted a cancer stem cell phenotype in nasopharyngeal cancer cells; however, it is not known if this was the result of ISG15 acting as a free or secreted protein or through ISGylation (71). ISG15 and ISGylation have also been shown to be increased in anaplastic thyroid cancer stem cells by flow cytometry (72). The investigators further showed that KPNA2 is stabilized by ISGylation and drives c-myc–mediated expression of pluripotency genes including *NANOG* and *OCT4*.

In contrast, Doherty *et al.* reported that IFNβ reversed stem cell properties in a model of triple negative breast cancer stem cells (73). However, a recent study demonstrated that type I interferon treatment of cancer cells increased the lysine specific demethylase KDM1B, which epigenetically reprograms cancer cells to generate cancer stem cells exhibiting properties of immune evasion and chemoresistance (29). In addition to mediating stem cell generation, interferons can act to slow cell proliferation and trigger apoptosis. The activation of apoptosis is mediated by caspase activation involving downstream ISGs as well as through activation of PI3K and mTOR by Janus kinase (74, 75).

Consistent with previous studies, we found that FTE-194 cells readily form tight spheroids (37) and express transcripts for multiple pluripotency genes. The finding that treatment with intracellular Poly I:C diminished spheroid-forming capacity in parental and UBA7-null cells, but not in ISG15-null cells, highlights an inhibitory role of ISG15 distinct from ISGylation. In fact, a more pronounced decrease in spheroid-forming capacity relative to parental cells appeared to occur in UBA7-null cells. One possibility is that loss of free ISG15 destabilizes USP18, leading to increased type I interferon signaling. Indeed, our experiments consistently demonstrate decreased levels of USP18 protein levels in ISG15-null cells, but not in UBA7-null cells. However, loss of ISG15 did not decrease spheroid-forming capacity in the absence of Poly I:C treatment. Assessment of several pluripotency associated gene transcript levels indicated a similar impact of Poly I:C in parental, ISG15-null and UBA7-null cells, with the exception of *NANOG*. Loss of ISG15 lead to increased transcript levels relative to parental cells, whereas loss of ISGylation lead to decreased transcript levels. Thus, ISGylation may act to support *NANOG* expression, whereas free ISG15 inhibits expression or transcript stability. Several studies have highlighted a role for NANOG in cancer initiation through enhancing STAT3 activity (76), increasing proto-oncogenic gene expression such as *YAP1* (77), and reprograming mitochondrial metabolism to favor fatty acid oxidation (78). Future studies are required to comprehensively explore the potential impact of loss of ISGylation on emergence of cancer stem cells within the fallopian tube.

In the present study, we found that loss of total ISG15 or ISGylation resulted in decreased cell migration and prevented intracellular Poly I:C from further inhibiting migration. This is consistent with studies indicating that ISG15 promotes migration and epithelial-to-mesenchymal cell transition (52, 53, 79). *SNAI1* is a direct target of IRF3, and viral infection as well as intracellular Poly I:C treatment have been shown to induce *SNAI1* and *SNAI2* expression (80). ISG15 has also been shown to interact with RAC1 to promote cell migration of oral squamous cancer cells, although this was independent of ISGylation (81).

**Figure 10:**
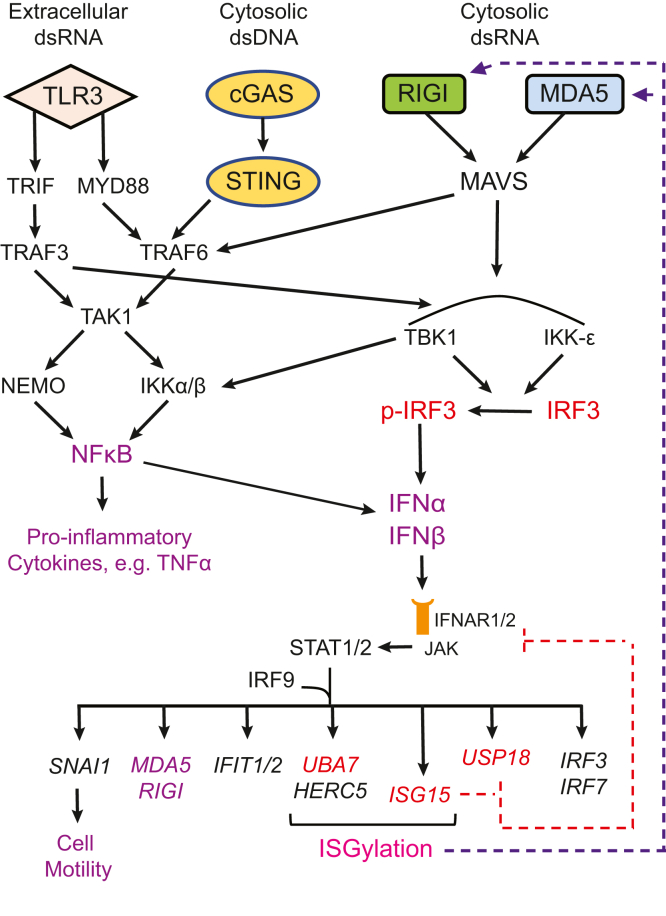
**Schematic model of the impact of ISGylation on proinflammatory signaling stimulated by intracellular dsRNA in FTE cells.** Intracellular Poly I:C is sensed by MDA5 or RIGI, which results in recruitment and aggregation of MAVS that facilitates constitutively expressed IRF3 phosphorylation by TBK1 or IKK-ε. Activated IRF3 induces expression and secretion of type I interferons, which bind to their plasma membrane receptors, IFNAR1/2, to result in phosphorylation of STAT1 and STAT2. The activated transcription factors partner with IRF9 and translocate to the cell nucleus to activate expression of IFN-stimulated genes. Among these are IRF3 and IRF7, which further promote IFN production, and genes involved in ISGylation, such as *ISG15*, *UBA7*, *HERC5*, and *USP18*, as well as numerous other immune related genes such as *IFIT1* and *IFIT2*, *MDA5*, *RIGI*, *cGAS,* and *SNAI1*. In addition to its action as an ISG15 deconjugase, USP18 acts to inhibit activation of STAT proteins by type I interferons, an activity that is promoted by free ISG15. Thus, free ISG15 and USP18 act to inhibit interferon signaling to limit the immune response. Aggregation of MAVS also leads to activation of NFκB signaling and downstream proinflammatory cytokines. In this study, we demonstrate that loss of ISGylation results in markedly decreased ability of intracellular dsRNA to activate IRF3 and NFκB signaling, and thus may be an attractive target to mitigate risk of HGSTOC in *BRCA1* mutation carriers. FTE, fallopian tube epithelial; HGSTOC, high-grade serous tubo-ovarian cancer; IFN, interferon; ISG, interferon-stimulated gene; Poly I:C, polyinosinic:polycytidylic acid.

Our previous work established an increased proinflammatory gene expression signature in the FTE of *BRCA1* mutation carriers compared to controls (13, 15) and highlighted a further increase in ISGs following exposure to periovulatory follicular fluid (13). Among these genes were members of the ISGylation pathway, most notably ISG15. Recent and emerging studies implicate activation of molecular pattern recognition signaling pathways in BRCA1-deficient breast and FTE cells as a cause of increased proinflammatory signaling. The data in this study demonstrate a role for ISGylation in promoting RIGI signaling resulting in increased activation of IRF3 and NFκB. Intact ISGylation appeared to result in stabilization of both MDA5 and RIGI, suggesting a likely additional possibility of enhanced MDA5 signaling (summarized in Fig. 10). These results support the idea that increased ISGylation in FTE cells, perhaps resulting from exposure to follicular fluid at the time of ovulation, could act to amplify proinflammatory signaling and contribute to a microenvironment conducive for carcinogenesis. Work is required to further test this possibility and determine if key enzymes specific to ISGylation, such as UBA7, would be suitable targets for ameliorating the risk for HGSTOC in BRCA mutation carriers.

## Experimental procedures

### Cell culture and reagents

Human immortalized nonmalignant FTE cell lines FTE-194 and FTE-246 (obtained from Dr R. Drapkin; University of Pennsylvania) and OE-E6/7 (obtained from Dr W.S.B. Yeung; The University of Hong Kong) were maintained in Dulbecco's modified Eagle's medium/F12 medium supplemented with 10% heat-inactivated fetal bovine serum, 100 U/ml penicillin, and 100 μg/ml streptomycin (Invitrogen). Cells were maintained in a humidified incubator at 37 °C with 5% CO_2_ atmosphere and used within 20 passages. Short tandem repeat analysis was used to verify human female derivation and cells tested negative for *mycoplasma* contamination.

Recombinant human IFNβ (R&D) and high molecular weight Poly I:C (InvivoGen) were dissolved in sterile water to a stock concentration of 56,000 IU/μl and 1 mg/ml, respectively. The stock solution aliquots were further diluted in culture media at time of use. dsDNA90 (4 μM) was generated by annealing 90 bp single-stranded sense and antisense DNA strands using sequences described by Abe and Barber (82).

### Generation of ISG15-null FTE-194 cells

*ISG15* was disrupted in FTE-194 cells using CRISPR-Cas9 and prime editing approaches. For the CRISPR-Cas9 approach (83), double-strand breaks within exons 1 and 2 were generated by introducing two pSpCas9(BB)-2A-GFP (Addgene; plasmid 48138) constructs resulting in the loss of 492 bp and insertion of a premature stop codon (Fig. S5*A*) as described by Merkert *et al.* (84). Cells were transfected using jetOPTIMUS transfection reagent (Polyplus) as per manufacturer’s instructions and transient GFP-expressing cells were isolated and clonally propagated. Clones were screened for loss of ISG15 by Western blot analysis (Fig. S5*B*). Disruption of *ISG15* was further verified in selected clones by PCR and gel electrophoresis to confirm the 492 bp gene deletion (Fig. S5*C*).

For the prime editing approach (85), an insert containing a unique *SnaBI* restriction site and premature stop codon was introduced within exon 2 of *ISG15* (Fig. S6*A*). The prime editing guide RNA (pegRNA) sequence for the edit target site (PE2) within exon 2 and the upstream opposite strand nicking site (PE3) within the intron region of the *ISG15* gene were selected with the pegFinder tool (http://pegfinder.sidichenlab.org/). For PE2 cloning, the oligonucleotide duplexes—including pegRNA spacer, pegRNA 3′-extension template, and SpCas9 single guide RNA (sgRNA) scaffold containing the appropriate overhangs—were annealed following phosphorylation of the scaffold sequences using T4 Polynucleotide Kinase (New England Biolabs). The annealed pegRNA duplexes and SpCas9 sgRNA scaffold were ligated with pU6-pegRNA-GG vector (Addgene; plasmid 132777) at the *BsaI* restriction site to generate the PE2-pegRNA plasmid. Correct ligation was sequence-verified. For the PE3 construct, the nicking sgRNA was annealed, phosphorylated, and inserted into a BPK1520 (Addgene; plasmid 65777) vector at the *BsmBI* site and sequence-verified. The pCMV-PE2-P2A-GFP (Addgene; plasmid 132776) and the PE2-pegRNA construct along with sgRNA-BPK1520 construct were cotransfected into FTE-194 cells using Lipofectamine 2000 (Invitrogen) as per manufacturer’s instructions. Transiently GFP-expressing cells were isolated and clonally propagated. Clones were screened for edited *ISG15* by *SnaBI* restriction digestion of PCR-amplified exon 2 and further verified by Western blot analysis (Fig. S6, *B* and *C*). Sequences used for prime editing generation of ISG15-null cells are listed in Table S1.

### Generation of UBA7-null FTE-194 cells

The *UBA7* gene was disrupted as described by Holthaus *et al.* (86) using a conventional CRISPR-Cas9 approach (Fig. S7*A*). gRNA oligos were annealed and inserted into pSpCas9(BB)-2A-GFP plasmid at the *BbsI* site and sequenced-verified. Cells were transfected using jetOPTIMUS transfection reagent (Polyplus) and transiently GFP-expressing cells were isolated and clonally propagated. Clones were screened for loss of UBA7 by Western blot analysis (Fig. S7*B*). To rescue the loss of UBA7, FTE-194 and FTE194-ΔUBA7c13 cells were seeded in 24-well plates at a density of 90,000 cells/well. FTE194-ΔUBA7c13 cells were transfected with UBA7 expression vector (Clone ID: OHuH04132; GeneScript) or empty vector (pcDNA3.1H^+^; Invitrogen) using Lipofectamine 2000. Forty-eight hours later, cells were transfected with or without 0.3 μg Poly I:C for 1 h.

### siRNA transfection

Cells were seeded in 6-well plates at a density of 400,000 cells/well. The following day, cells were transfected with 50 nM siRNA pool targeting MDA5 or 100 nM siRNA pool targeting RIGI (siMDA5 and siRIGI; Dharmacon) or control non-targeting siRNA (On-TARGET plus control siRNA; Dharmacon) using Lipofectamine 2000 72 h prior to harvesting. For IFNβ pretreatment, cells were treated with IFNβ (500 IU/ml) or vehicle (sterile water) 48 h after siRNA transfection. The cells were harvested 1 h after Poly I:C transfection or 2h after addition of naked Poly I:C.

### Poly I:C or dsDNA90 treatment

Cells were seeded into 24-well plates at a density of 90,000 cells/well and treated with IFNβ (500 IU/ml) or vehicle. Poly I:C or dsDNA90 transfection was performed 20 h later using Lipofectamine 2000. For studies using naked Poly I:C, cells were seeded into 6-well plates at a concentration of 400,000 cells/well. The following day, cells were treated with IFNβ (500 IU/ml) or vehicle and 20 h later, naked Poly I:C or vehicle (sterile water) was added. The time and concentration of Poly I:C and dsDNA90 transfection or naked Poly I:C addition are as indicated for individual experiments.

### Real-time qPCR

Total RNA was extracted using RNeasy Plus Universal Mini Kit (Qiagen) and reverse transcribed using SuperScript VILO cDNA synthesis Kit (Invitrogen) as per manufacturer’s instructions. Primers used are listed in Table S2. qPCR was performed on a CFX OPUS 96 Real-time PCR system (Bio-Rad) with SYBR Green PCR Master Mix (Bio-Rad). The cycling conditions were 95 °C for 10 s, 95 °C for 15 s, and 60 °C for 60 s for 40 cycles. A dissociation curve was performed at the end of the PCR cycle to confirm single product amplification. Data were analyzed using the ΔCCT method with the target gene C_T_ value normalized to the geometric mean of housekeeping genes (*GAPDH*, *B2M,* and/or *18S rRNA*) as described (87, 88).

### Western blotting

Cells were lysed in radio immunoprecipitation buffer (50 mM Hepes, 150 mM NaCl, 1% Triton X-100, 0.1% SDS, 1% sodium deoxycholate) containing Complete protease and phosphatase inhibitor cocktails (Roche Diagnostics) for 30 min at room temperature. Total cell lysates were collected by centrifugation at 17,750*g* for 15 min at 4 °C. Total protein concentration was determined using a Pierce Protein Bicinchoninic Acid assay kit (Thermo Fisher Scientific) as per manufacturer’s instructions.

Protein extracts (10–40 μg) were resolved by SDS-PAGE (10%–15% gels), transferred to polyvinylidene flouride membrane and incubated overnight with primary antibody (listed in Table S3) at 4 °C. The blots were washed with either PBS with 0.1% (v/v) Tween 20) or tris-buffered saline with 0.1% (v/v) Tween 20 for 30 min at room temperature and probed with horseradish peroxidase–conjugated goat anti-mouse or anti-rabbit antibody (both at 1:1000; Cell Signaling Technology) for 1 h at room temperature. Immunoreactive bands were detected by enhanced chemiluminescence (Bio-Rad), and density was quantified using Image lab (Bio-Rad). For quantitation of ISGylation, the density of the lane (20 kDa to 250 kDa) was measured.

### Culture growth assay

Cells were seeded in 96-well plates at a density of 2500 cells/well and treated with IFNβ (500 IU/ml) or vehicle 24 h later. For experiments including Poly I:C treatment, cells were seeded at 90,000 cells/well in 24-well plates and transfected with or without Poly I:C (0.3 μg) 48 h later. One hour later, cells were transferred to a 96-well plate (2500 cells/well). Relative cell number was determined 0, 24, 48, and/or 72 h later as indicated for individual experiments using an XTT dye reduction assay. XTT (Invitrogen), to a final concentration of 50 μg/ml, was added to the wells and absorbance at 450 nM was determined 3 h later.

### Cell migration

Cell migration was assessed using a scratch-wound assay. Cells were seeded in 24-well plates at high density (180,000 cells/well) and transfected with or without Poly I:C (0.3 μg) 24 h later. The confluent monolayer was scratched with a sterile 1 ml pipette tip 1h after transfection, washed twice with PBS and the medium was replaced with serum-free medium. Images of the wound were taken using an inverted microscope at the time of wounding (T = 0 h) and 22 h later. The area of the open wound was measured using Image J software (https://imagej.net/downloads) and expressed as [open area at T = 22 h/open area at T = 0 h] × 100.

### Spheroid formation

Cells seeded in 24-well tissue culture plates were transfected with or without 0.3 μg Poly I:C. Cells were trypsinized 1 or 3h after transfection and seeded 25,000 cells/well (Fig. 9) or 2500 cells/well (Fig. S4) into 96-well low-attachment tissue culture plates (Corning). Spheroids were photographed 24, 48 and 72 h after seeding.

### Statistical analysis

Data are expressed as the mean ± SEM of at least three independent experiments. A Grubbs test was used to identify outliers. Data were analyzed by two- or three-way ANOVA, followed by Holm–Sidak multiple comparison test as appropriate. Statistical significance was determined at *p* < 0.05. GraphPad Prism (https://www.graphpad.com; version 9.2.0) was used for data analysis.

## Data availability

All data are provided in the article.

## Supporting information

This article contains supporting information.

## Conflict of interest

The authors declare that they have no conflicts of interest with the contents of this article.
