# Supplementary material for: ISGylation enhances dsRNA-induced interferon response and NFκB signaling in fallopian tube epithelial cells
Source: J Biol Chem. 2024 Aug 17;300(9):107686. doi: 10.1016/j.jbc.2024.107686 (PMC11418117; doi:10.1016/j.jbc.2024.107686)
Supplement: Supporting Tables and Figures [file mmc1.pdf]

SUPPORTING INFORMATION

Title: ISGylation enhances double-stranded RNA-induced interferon response and NFκB signaling in fallopian tube epithelial cells

Authors: Vidushi Madaan[1,2], Alexandra Kollara[1], David Spaner[2,3], and Theodore J. Brown[1,2,4*]

List:

Table S1: List of ISG15 oligos used to generate ISG15-null cells by Prime Editing.

Table S2: List of primers used for qRT-PCR.

Table S3: List of antibodies used in the study.

Fig. S1: Optimization of dose and time of dsDNA90 and Poly I:C transfection or addition on IRF3 activation.

Fig. S2: Loss of ISGylation does not impact activation of IRF3 or RELA by naked Poly I:C.

Fig. S3: Optimization of parameters for siRNA targeting MDA5 or RIGI in FTE-194 cells.

Fig. S4: Loss of ISG15 causes tight spheroid formation and increased *NANOG* transcript levels.

Fig. S5: Generation of ISG15-null FTE-194 cells by CRISPR-Cas9.

Fig. S6: Generation of ISG15-null FTE-194 cells by Prime Editing.

Fig. S7: Generation of UBA7-null FTE-194 cells by CRISPR-Cas9.

Table S1: List of ISG15 oligos used to generate ISG15-null cells by Prime Editing.

**PE2 pegRNA**	Top: 5'-CACCGCCGCCAGCATCTTCACCGTCGTTTTAGA-3'<br>Bottom: 5'-TAGCTCTAAAACGACGGTGAAGATGCTGGCGGC-3'
**pegRNA 3'-extension template**	Top: 5'-GTGCGGGACCTTACGTAGGTGAAGATGC-3'<br>Bottom: 5'-AAAAGCATCTTCACCTACGTAAGGTCCC-3'
**PE3 sgRNA**	Top: 5'-CACCGTGTGTGGTGGGCCTGGGGC-3'<br>Bottom: 5'-AAACGCCCCAGGCCCACCACACAC-3'

Table S2: List of primers used for qRT-PCR.

Gene ID	Primer Sequence	NCBI accession #	Amplicon Size
ISG15	F: GAGAGGCAGCGAACTCATCT<br>R: CAGGGACACCTGGAATTCGTT	NM_005101.4	127 bp
TNFα	F: CCCAGGGACCTCTCTCTAATC<br>R: ATGGGCTACAGGCTTGTCACT	NM_000594	84 bp
PTGS2	F: GTTCCCACCCATGTCAAAAC<br>R: AATTCCGGTGTTGAGCAGTT	NM_000963.4	112 bp
IFNB1	F: TGCTCTCCTGTTGTGCTTCT<br>R: TTCAATTGCCACAGGAGCTT	NM_002176.4	111 bp
CCL5	F: GCCTCTCCCACAGGTACCAT<br>R: GGTGTGGTGTCCGAGGAATA	NM_002985.3	113 bp
GAPDH	F: CGAGCCACATCGCTCAGA<br>R: AGTTAAAAGCAGCCCTGGTGA	NM_002046	95 bp
B2M	F: CTCCGTGGCCTTAGCTGTG<br>R: TTGGAGTACGCTGGATAGCCT	NM_004048	68 bp
18S	F: AGAAACGGCTACCACATCCA<br>R: CCCTCCAATGGATCCTCGTT	XR_007090847	138 bp
NANOG	F: AGAACTCTCCAACATCCTGAACCT<br>R: TGCCACCTCTTAGATTTCATTCTCT	NM_024865.4	84 bp
OCT4	F: CCGTGAAGCTGGAGAAGGAG<br>R: GCAGATGGTCGTTTGGCTGA	NM_002701.6	194 bp
SOX2	F: CCCCTTTATTTTCCGTAGTTGTATTT<br>R: TGGATTCTCGGCAGACTGATT	NM_003106.4	73 bp
ALDH1	F: TCAAAGAAGCTGCCGGGAAA<br>R: GTCCAAGTCGGCATCAGCTA	NM_000689.5	95 bp
CD44	F: TGCCGCTTTGCAGGTGTATT<br>R: CCGATGCTCAGAGCTTTCTC	NM_000610.4	137 bp
SSEA4 synthase	F: TGGACGGGCACAACTTCATC<br>R: GGGCAGGTTCTTGGCACTCT	NM_006927.4	119 bp

Table S3: List of antibodies used in the study.

Antibody	Supplier	Catalog #	Species	Dilution for
p-S386 IRF3	Cell Signaling	37829	Rabbit	1:500
IRF3	Santa Cruz	sc-33641	Mouse	1:500
ISG15	Santa Cruz	sc-166755	Mouse	1:500
GAPDH	Cell Signaling	2118	Rabbit	1:1000
HSP90	BD Biosciences	610418	Mouse	1:3000
MDA5	Cell Signaling	5321	Rabbit	1:500
p-S536 NF-κB	Cell Signaling	3033	Rabbit	1:1000
NF-κB p65	Cell Signaling	6956	Mouse	1:1000
RIGI	Cell Signaling	3743	Rabbit	1:500
UBA7	Cell Signaling	61266	Rabbit	1:1000
USP18	Cell Signaling	4813	Rabbit	1:1000

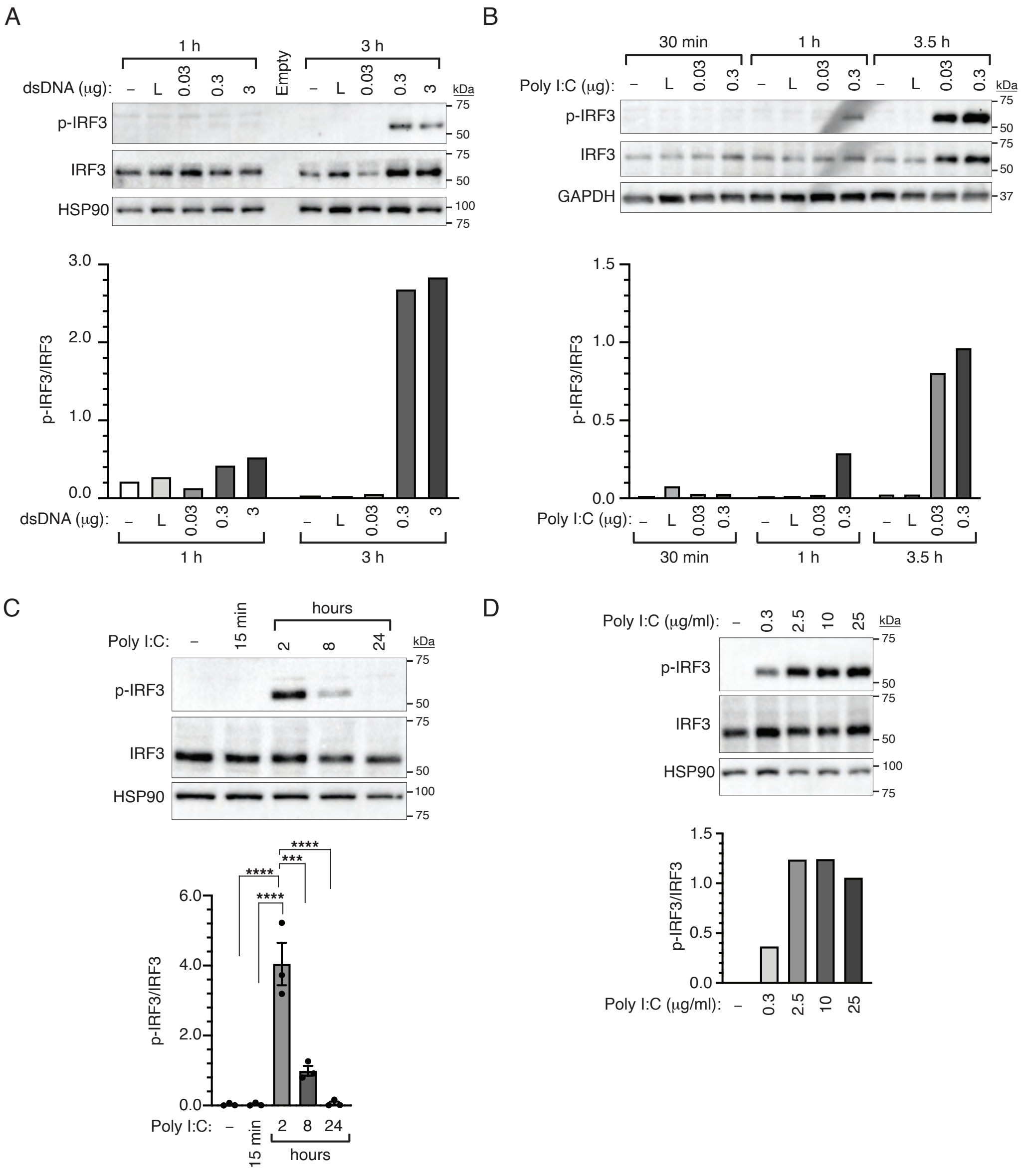

Fig. S1: Optimization of dose and time of dsDNA90 and Poly I:C transfection or addition on IRF3 activation. Phospho-IRF3 (p-IRF3) levels following transfection of dsDNA90 *(panel A)* or Poly I:C (*panel B*) in FTE-194 cells at the indicated doses and times. *C,* p-IRF3 levels at various times following addition of 25 µg/ml naked Poly I:C to the culture medium. Bars represent the mean ± SEM of three independent experiments. ***p≤0.001, ****p≤0.0001 as determined by Holm-Sidak multiple comparison test following two-way ANOVA. *D,* p-IRF3 levels at 2 h following addition of varying doses of naked Poly I:C to the culture medium. L, lipofectamine alone; Poly I:C, polyinosinic:polycytidylic acid.

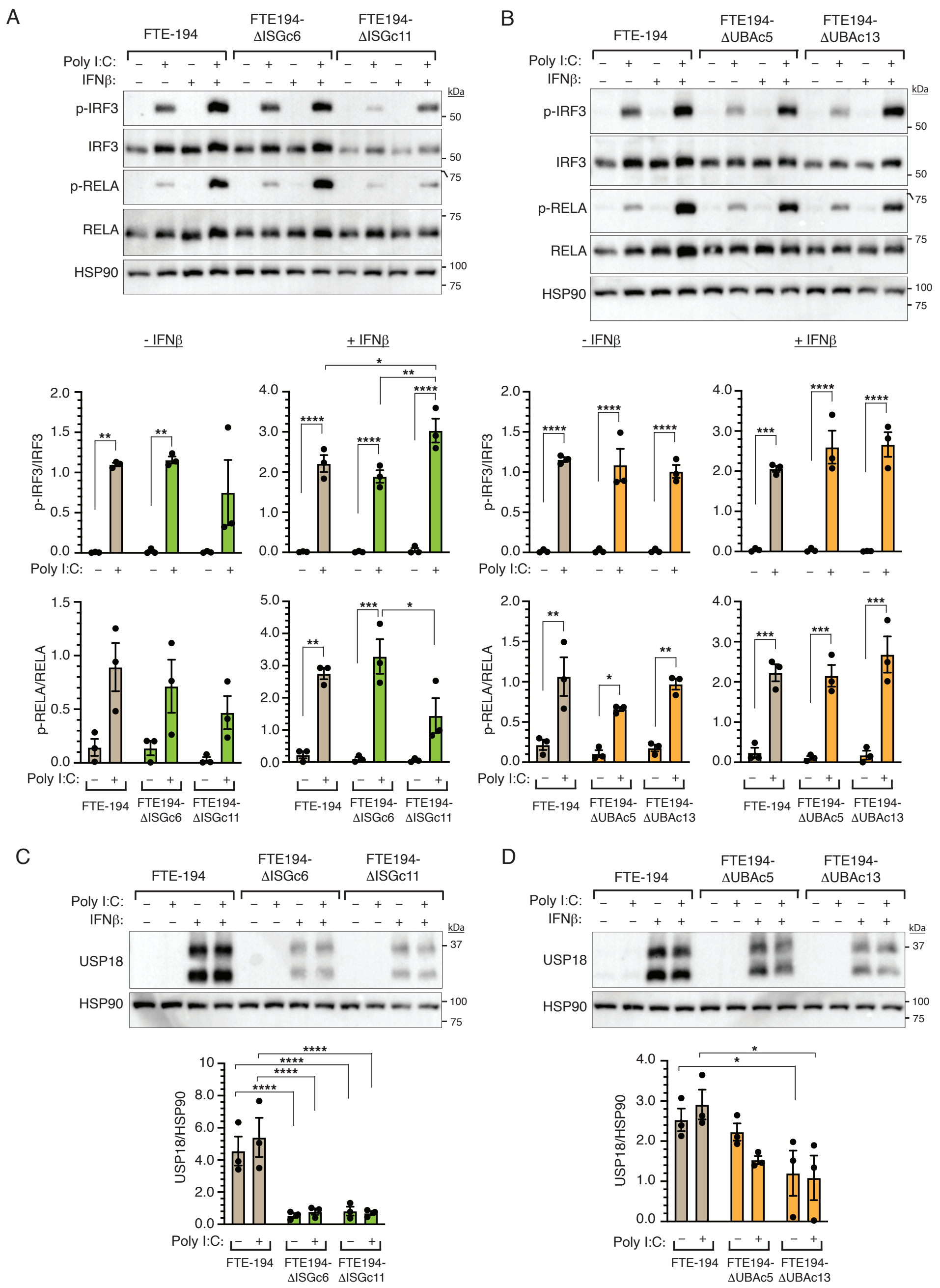

Fig. S2: Loss of ISGylation does not impact activation of IRF3 or RELA by naked Poly I:C. *A and B*, representative Western blots and bar graphs summarizing p-IRF3 and p-RELA levels in parental FTE-194 cells and two clonally selected ISG15-null cell sublines (*panel A*) or in parental FTE-194 cells and two clonally selected UBA7-null cell sublines (*panel B*). *C and D*, representative Western blots and bar graphs summarizing levels of USP18 in parental and ISG15-null (*panel C*) and UBA7-null (*panel D*) cells pretreated with IFNβ. Cells were pretreated with or without 500 IU/ml IFNβ and exposed to 0.3 μg Poly I:C for 2 h before harvesting. HSP90 was used as a loading control. Bars represent the group mean ± SEM of three independent experiments. *p≤0.05, **p≤0.01, ***p≤0.001, ****p≤0.0001. IFN, interferon; Poly I:C, polyinosinic:polycytidylic acid.

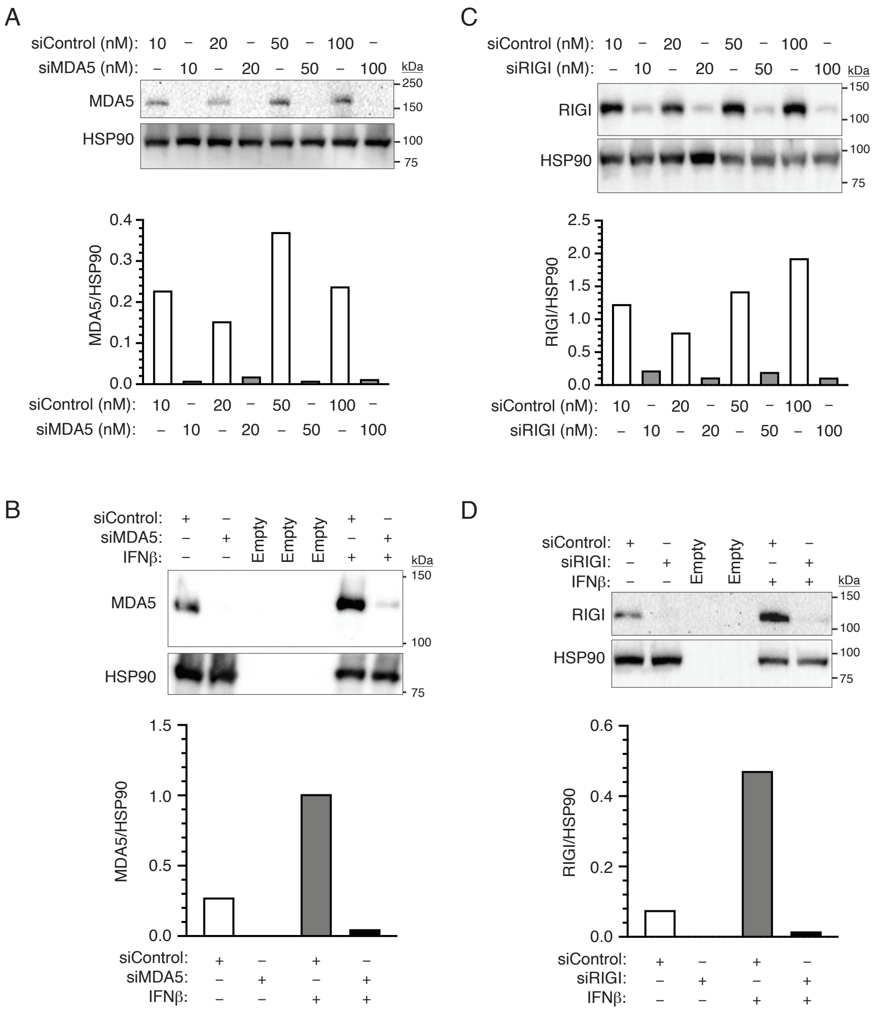

Fig. S3: Optimization of parameters for siRNA targeting MDA5 (*panels A and B*) or RIGI (*panels C and D*) in FTE-194 cells. *A and C*, Cells were transfected with varying concentrations of pooled MDA5 siRNAs (*panel A*) or pooled RIGI siRNAs (*panel C*) or nontargeting control siRNA and the levels of MDA5 or RIGI protein were assessed 72 h later. *B and D,* cells were transfected with 50 nM pooled MDA5 siRNAs (*panel B*) or 100 nM pooled RIGI siRNAs (*panel D*) or control siRNA and treated with or without 500 IU/ml IFNβ 48 h later. Cells were harvested 20 h after IFNβ treatment and MDA5 and RIGI levels were assessed by Western blot analysis. IFN, interferon.

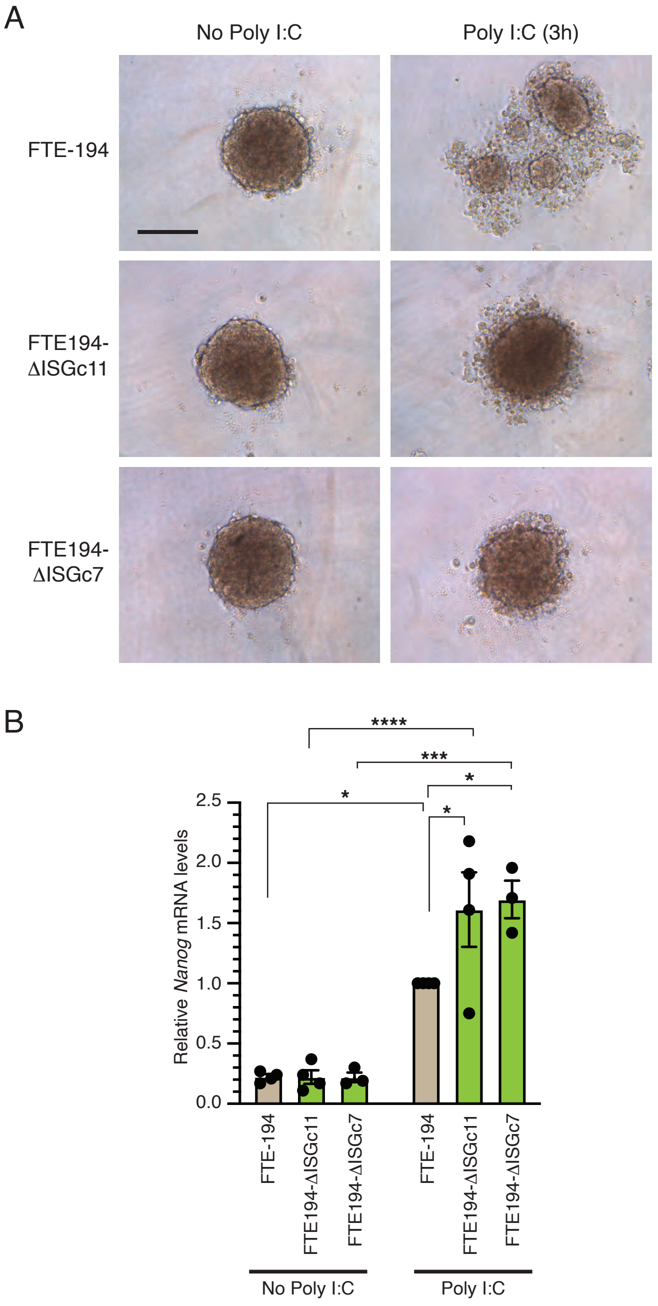

Fig. S4: Loss of ISG15 causes tight spheroid formation and increased *NANOG* transcript levels. *A,* representative images of spheroids formed by parental, and two clonally selected ISG15-null cell sublines transfected with or without 0.3 µg Poly I:C for 3 h. Identical results were obtained in three independent experiments, each with 2 to 3 replicates. The scale bar represents 100 µm. *B,* relative transcript levels of *NANOG* measured by qPCR in parental, and two clonally selected ISG15-null cell sublines after transfection with or without 0.3 µg Poly I:C for 3 h. Values are relative to levels measured in parental cells treated with Poly I:C. Bars represent the group mean ± SEM of 3 to 4 independent experiments. Significant group comparisons as determined by ANOVA followed by Holm-Sidak's multiple comparison testing are shown. *$p \leq 0.05$, ***$p \leq 0.001$, ****$p \leq 0.0001$. Poly I:C, polyinosinic:polycytidylic acid.

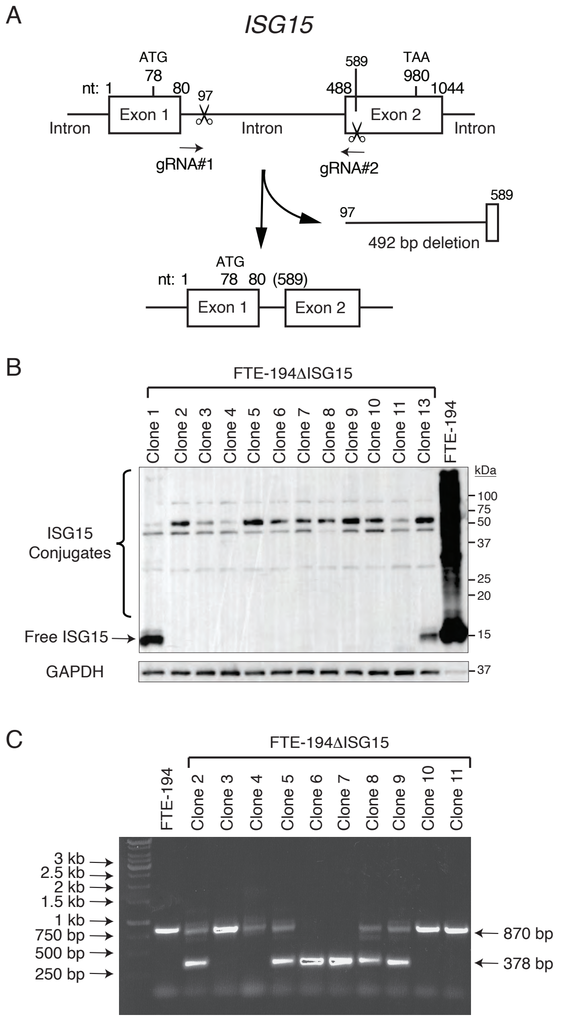

Fig. S5: Generation of ISG15-null FTE-194 cells by CRISPR-Cas9. *A,* schematic showing the two sites of double-strand cuts generated within the genomic sequence of ISG15 by transfection of cells with two pSpCas9(BB)-2A-GFP constructs. This results in the loss of a 492 bp fragment from the gene resulting in generation of an early stop codon in transcripts. *B*, representative Western blot showing the loss of free and conjugated ISG15 in several single cell-derived clones following treatment with 1000 IU/ml IFNβ 24 h before harvesting. *C*, a representative agarose gel showing the loss of the 492 bp fragment from the ISG15 alleles in clone 6 and 7 cells. gRNA, guide RNA.

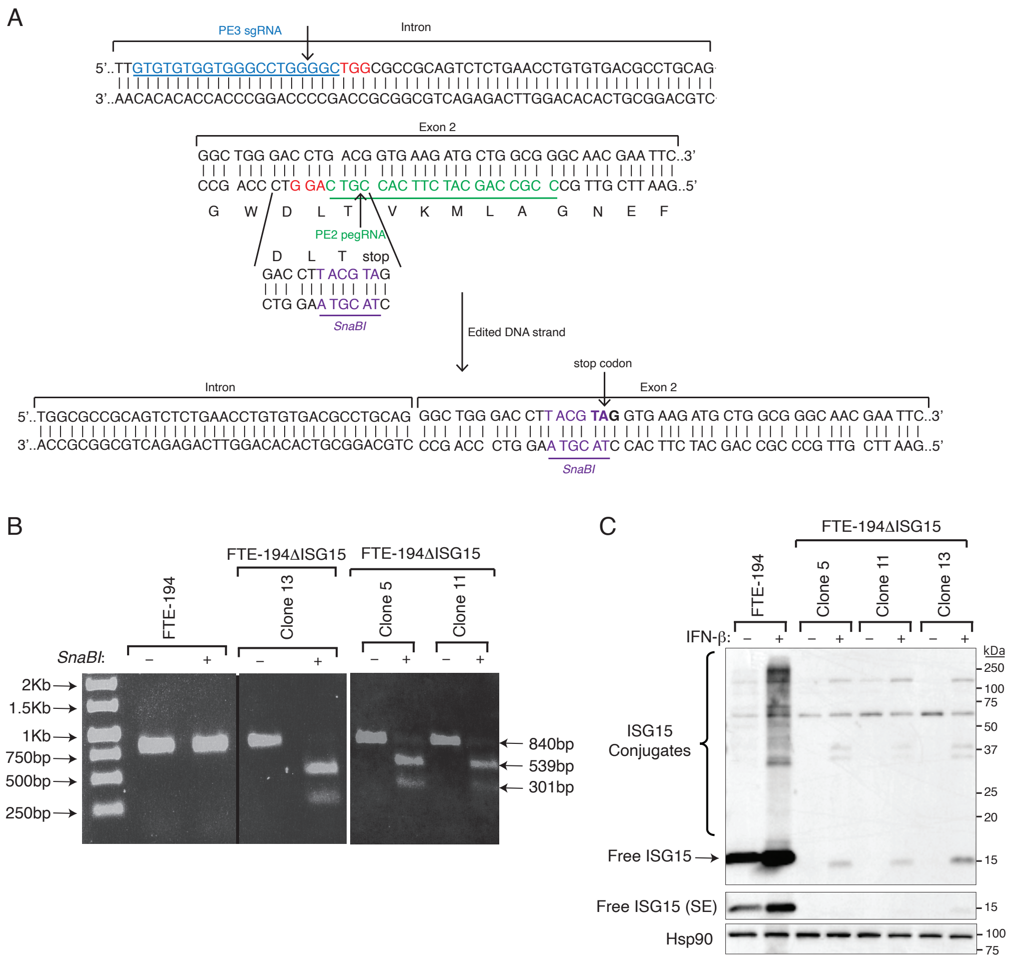

Fig. S6: Generation of ISG15-null FTE-194 cells by Prime Editing. *A,* schematic showing the site of the edit generated by the PE2 construct to incorporate a premature stop codon and a unique *SnaBI* restriction site within exon 2. To enhance efficiency of the edit, a nick in the upstream sequence of the opposing strand was generated with a PE3 construct. *B,* a representative agarose gel showing the incorporation of the *SnaBI* restriction site in three single cell-derived clones. An 840 bp fragment of the targeted editing site of ISG15 was generated by PCR and subjected to *SnaBI* digestion. The presence of the cleaved products and absence of an 840 bp fragment served as an indication of disruption of both ISG15 alleles. *C,* representative Western blot showing the loss of free and conjugated ISG15 in the three clones following treatment with 1000 IU/ml IFNβ or vehicle 24 h before harvesting. SE, short exposure; IFN, interferon; sgRNA, single guide RNA; pegRNA, prime editing guide RNA.

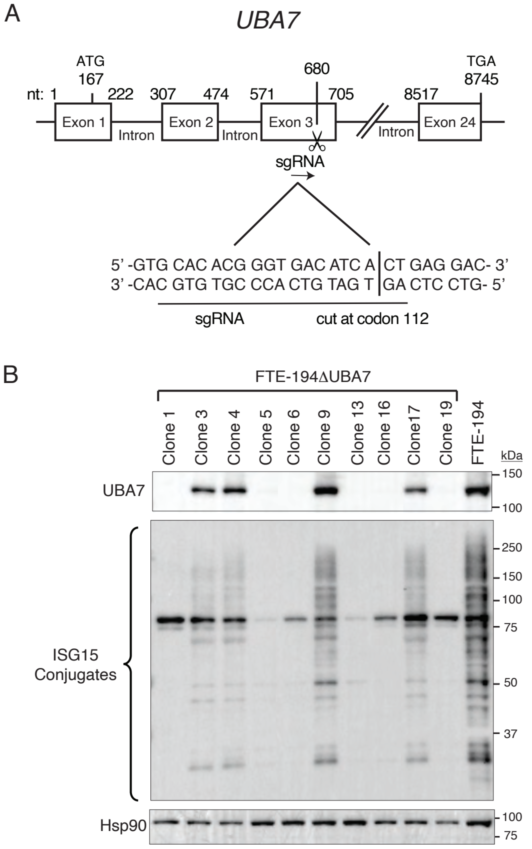

Fig. S7: Generation of UBA7-null FTE-194 cells by CRISPR-Cas9. *A,* schematic showing the site of the double strand cut generated within the genomic sequence of UBA7 by transfection of cells with the pSpCas9(BB)-2A-GFP construct. This disruption results in errors generated by non-homologous end joining repair, resulting in the incorporation of a premature stop codon. *B,* representative Western blot showing the loss of UBA7 in several single cell-derived clones and the loss of ISGylation following treatment with 1000 IU/ml IFNβ 24 h before harvesting. sgRNA, single guide RNA.
